# Application of Networking Approaches to Assess the Chemical Diversity, Biogeography, and Pharmaceutical Potential of Verongiida Natural Products

**DOI:** 10.3390/md19100582

**Published:** 2021-10-18

**Authors:** James Lever, Robert Brkljača, Colin Rix, Sylvia Urban

**Affiliations:** 1 School of Science (Applied Chemistry and Environmental Sciences), RMIT University, GPO Box 2476, Melbourne, VIC 3001, Australia; james.lever@rmit.edu.au (J.L.); colin.rix@rmit.edu.au (C.R.); 2 Monash Biomedical Imaging, Monash University, Clayton, VIC 3168, Australia; Robert.brkljaca@monash.edu

**Keywords:** network analysis, cheminformatics, verongiida sponges, natural products, in silico mapping

## Abstract

This study provides a review of all isolated natural products (NPs) reported for sponges within the order Verongiida (1960 to May 2020) and includes a comprehensive compilation of their geographic and physico-chemical parameters. Physico-chemical parameters were used in this study to infer pharmacokinetic properties as well as the potential pharmaceutical potential of NPs from this order of marine sponge. In addition, a network analysis for the NPs produced by the Verongiida sponges was applied to systematically explore the chemical space relationships between taxonomy, secondary metabolite and drug score variables, allowing for the identification of differences and correlations within a dataset. The use of scaffold networks as well as bipartite relationship networks provided a platform to explore chemical diversity as well as the use of chemical similarity networks to link pharmacokinetic properties with structural similarity. This study paves the way for future applications of network analysis procedures in the field of natural products for any order or family.

## 1. Introduction

Marine sponges (phylum Porifera, Grant 1836 [1]) are benthic invertebrates that play host to a rich and diverse number of microbial symbionts. Marine sponge holobionts or their symbionts have been the source of an extraordinary number of biologically important chemical compounds, termed natural products (NPs). The compounds isolated have generally been of high chemical diversity and are often unique, not only in the structures that they exhibit, but also in the broad range of biological activities that they display [2]. This array of bioactivity has sustained great interest in marine sponges as a source of compounds with pharmaceutical potential.

Contemporary NP research is centred around the hypothesis that targeting organisms or sampling sites with high biodiversity will lead to more chemically diverse compounds, and thus a larger variety of bioactivities [3,4,5]. The understanding of biogeographical and ecological diversity, and the distribution trends of organisms that produce NPs, can be informative for future isolation efforts [6,7].

To date, the study of biogeographical trends which influence sponge NP production has been hindered by the dynamic nature of sponge taxonomy, particularly those within Verongiida Bergquist, 1978 [8], because the primary diagnostic tool is the structure and architecture of the laminated fibres. This, together with both the tendency of these organisms to occur in different forms under different environmental pressures, due to natural morphological plasticity, and the presence of diverse microbial symbionts, can create difficulty when deciding on the origin of compounds that are isolated from marine sponges. With more widespread and accurate data having been accumulated regarding taxonomy and the distribution of NPs across many families, the task of describing NP diversity and distribution is becoming a more achievable one.

A large body of work exists in the literature documenting and reviewing NPs isolated from specific genera within the order Verongiida [9,10,11,12,13]. This distinct marine sponge order is differentiated both phylogenetically and morphologically from other sponge orders [14]. The Verongiid sponges lack a mineral skeleton, displaying instead a heavily collagenous mesohyl which obtains shape and structure from spongin fibres that exhibit a granulated “pith” interior, together with a laminated “bark” exterior [14,15]. This marine sponge order has seen particular interest from the NP community over the past 50 years, due in part to the large number of bioactive bromotyrosine alkaloids (BTAs) that they produce [12]. BTAs from Verongiida show significant chemical diversity as a class, and provide effective chemical defence for these sessile invertebrates against predation [16,17] and fouling organisms [18,19].

Whilst these compounds are not exclusive to Verongiida [20], they do occur in greater quantities and present more structural variants within this order than any other. Given the taxonomic spread and geographic ubiquity of BTAs across this order, it is clear why they are considered by many as a significant taxonomic marker for Verongiida sponges [21,22,23].

BTAs represent a compound class of interest due to their chemical diversity as well as their propensity for wide ranging bioactivity [9,10,11,12]. Notable examples include the disulphide-linked psammaplins, first isolated from an unidentified specimen of *Psammaplysilla* Keller, 1889 (= *Pseudoceratina* Carter, 1885), in 1987 [24]. These compounds have inspired further studies of Verongiid sponges as well as the design of synthetically targeted anti-cancer drug libraries [25,26,27]. The BTA compounds from the Verongiid sponges show enormous pharmaceutical potential, with many viewed as being promising targets within the preclinical pipeline. Preclinical assays on BTAs have highlighted many candidates for antimalarial [28,29], antibacterial [30,31,32,33], antiprotozoal [30,34], anticoagulant [28,35,36] as well as potential central nervous system drugs [30,31,37,38]. This significant and broad-spectrum activity has provided much impetus to further study this order of sponge and its associated NPs.

Much debate has ensued regarding the origin of these NPs, with putative evidence suggesting contradicting theories of host vs. symbiont origins from Verongiid sponges [39,40,41,42]. It has even been suggested that the biogenetic origins of these compounds begin within sponge cells, followed by the translocation of intermediates for further biosynthetic transformation performed by symbiont microbes [43]. Whichever the case, it is a process that is poorly understood. Multi-omic related work on Verongiid sponges has shown a correlation between the microbial and metabolic architectures of a range of species sampled from differing locations [39]. This assessment implies that despite the differing core microbiomes of varying taxa of Verongiid sponges, there is chemical consistency when comparing species from different locations. Moreover, this aligns with the current understanding of the core microbiome as being highly species-dependent across differing geospatial and temperature gradients [44,45,46,47,48]. However, species specificity has not brought us any closer to understanding the origin of NPs from the Verongiid sponges; instead, it has highlighted that these organisms exist in a complex mutualistic ecosystem.

The geospatial and taxonomic conservation of microbiome architectures provides an opportunity to better understand the biogeographic and chemotaxonomic distribution of Verongiida NPs. With approximately 633 NPs reported from over 43 different species in the literature, there appears to be a suitable number of compounds with a wide enough taxonomic spread of species to provide a solid foundation on which to base an understanding of any trends in the distribution of NPs.

The goal of this data mining exercise was to characterise the geographical distribution of all NPs produced by sponges within the order Verongiida, highlighting key trends that may assist NP isolation efforts in the future. The work also addresses the distribution of NPs across different genera within the order Verongiida to identify possible chemotaxonomic or biosynthetic trends. Finally, the predicted structural similarities and pharmaceutical activity of these NPs is discussed, utilising network analysis methodologies.

## 2. Results and Discussion

In total, 215 papers were surveyed, which reported NPs that had been isolated from four families (43 species) of Verongiid sponges across approximately 126 geographical locations (see Supporting Information S1 for full reference list). Prior to this current study, reviews had been published on the genus *Aplysina* Nardo, 1834 [49], and its associated NPs in 2011 [10] and 2015 [11]. These reviews focused primarily on the listing and reporting of ^13^C NMR data and biological activity, as well as providing some insight into the proposed biosynthesis of some BTA compounds. In 2019, a review was also published on the genus *Suberea* Bergquist, 1995 [50], including compound lists as well as bioactivity and proposed biosynthesis [9]. A 2005 review documented the bioactivity and biosynthesis of the marine BTA derivatives as a compound class [12].

To date, there remains no review that encompasses the entire order of Verongiida. The present work rectifies this situation and focusses on NPs reported between the period of 1960 to May 2020. Compound types that have been described from this order of marine sponges are largely comprised of BTAs, including spiroisoxazolines (SIA) existing in both mono- and bis-configurations (mSIA and bSIA), spirooxepinisoxazolines, brominated phenolics, dibromocyclohexadienes, verongiabenzenoids, verongiaquinols, brominated oximes, oxime disulfides, bromotyramines (BT), bromotyramine oximes (BTOx), bastadins and hemibastadins. Further compounds that are not associated with the BTA biosynthetic route which are also found in the order, include hydroquinones, pyrroles, quinolines, guanidine alkaloids, indole alkaloids, benzonaphthyridines, benzofurans isoprenoids, sesterterpenoids, sesquiterpenoids, merosesquiterpenoids and macrolides.

### 2.1. Biosynthesis and Distribution of BTAs

**Figure 1:**
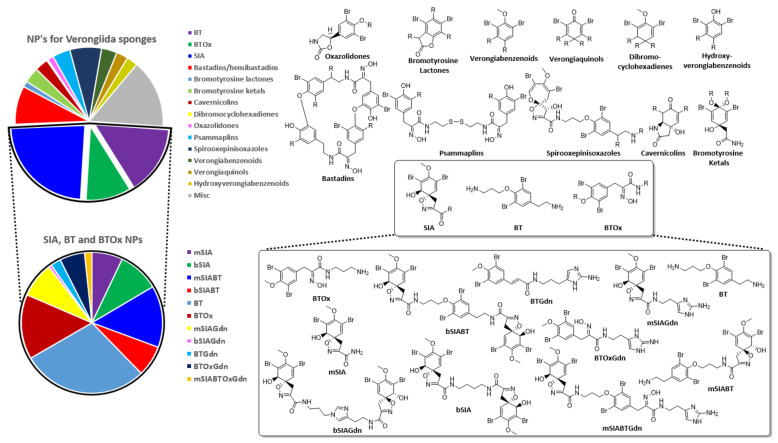
Classes of SIA, BT and BTOx. Mono spiroisoxazolines (mSIA), bis spiroisoxazolines (bSIA), bis spiroisoxazoline bromotyramine (bSIABT), mono spiroisoxazoline bromotyramine (mSIABT), mono spiroisoxazoline guanidine (mSIAGdn), bromotyramine (BT), bromotyramine oxime (BTOx), bis spiroisoxazoline guanidine (bSIAGdn), mono spiroisoxazoline bromotyrmamine oxime guanidine (mSIABTOxGdn), bromotyramine guanidine (BTGdn), bromotyramine oxime guanidine.

The SIA and BT classes form the basis of many sub classes of BTA including a large array of mono- and bis- spiro isomers. In addition, both SIAs and BTs are incorporated to create some of the higher molecular weight compound classes (Figure 1). Mono- and bis-SIA compounds are also observed together with BTs that form end groups using cyclised guanidine (Gdn) expressed as either imidazole, amino imidazole or imidazoline. These three classes, SIA, BT and BTOx, represent approximately 48% of all the NPs reported for Verongiida sponges and appear to be the major biosynthetic outcomes for these organisms. The SIA, BT and BTOx units are then utilised as the building blocks to create many more diverse structures, somewhat reminiscent of combinatorial chemistry (Figure 1 and Table 1). Approximately 18% of SIAs, BTs and BTOxs incorporate Gdn into their structures, resulting in either imidazole, imidazoline, imidazole amine or non-cyclic Gdn functionalities. Biosynthetically, these groups are often found as chain-terminating entities, except in the rare situations when Gdn can be found to reside between two SIA head groups. In this situation, the NH group of an imidazole ring provides an attachment point for another SIA head group.

SIAs appear to be the only spiro class of NP created by Verongiid sponges expressed with bis configurations. A bis spirooxepinisoxazoline has yet to be observed, despite the two classes supposedly originating from the same arene oxide intermediate. The formation of bis compounds would appear to be exclusive to the spirocyclohexadiene structure shown by SIAs. Conversion of the epoxide intermediate to either an SIA or a spirooxepinisoxazoline has been postulated to be enantioselective, rather than enantiospecific, as both (+) and (−) SIAs have been reported in differing quantities, suggesting enantiodivergence from the intermediate epoxide [51,52].

The distribution of these NPs (Table 1) across the order of Verongiida reflects that the two most studied genera, *Aplysina* and *Pseudoceratina* Carter, 1885 [53], have a wide variety of compound classes. Both genera have many species with reported bSIA and mSIA compounds, which are indicative of the presence of compounds such as aerothionin, homoaerothionin and purealidin R or purpuroacetic acid, respectively. *Aplysina* differs from *Pseudoceratina* in the production of compounds that possess Gdn derived moieties. *Aplysina* species appear to always express mSIAGdns, often in the form of aerophobin compounds, but only *A. archeri* (Higgin, 1875) [54] and *A. lacunosa* (Lamarck, 1814) [55] have been reported with other Gdn compounds. Comparatively, species of *Pseudoceratina* sponges show more complex chemistry with a wider variety of SIA/BT/Gdn combinations. The absence of Gdn also extends to the genus *Ianthella* Gray, 1869 [56], which has also displayed an apparent absence of the BTOx class of NP. This probably arises from the apparent tendency for *Ianthella* sponges to produce hemibastadins from their oxime bromotyramines prior to any O-methylation, which then acts as the precursor for the macrocyclic bastadins. This absence of BTOx compounds (other than hemibastadins) is informative, as they seem to produce the required BT precursors for the BTOx compounds that are largely absent, suggesting that the hemibastadins are the preferential biosynthetic route for *Ianthella*. The scarcity of SIAs reported for *Ianthella* indicates significant biogenetic divergence of this genus from the remainder of the Verongiid sponges, which is supported by its unique mesohyl biology and differentiation within Verongiida in the family Ianthellidae Hyatt, 1875 [57].

The current literature also shows an apparent absence of low molecular weight verongiabenzenoids, dibromocyclohexadienes and verongiaquinols within species of *Ianthella*. This evidence tends to contradict a postulate that no SIAs are observed due to biotransformation of these higher molecular weight compounds to lower molecular weight derivatives via the hypothesised wound induced chemical defence process. 

This same observation could be made for both *Hexadella* Topsent, 1896 [58] and *Aplysinella* Bergquist, 1980 [59]; however, these two genera are far less well-studied than *Aplysina*, *Pseudoceratina* and *Ianthella*. Despite this lack of SIAs for *Aplysinella* and *Hexadella,* it is still significant to note that only *Hexadella* sp. and *Aplysinella* sp. produced SIA compounds, while the remaining species either produced BTs or BTOx type compounds (Table 1). *Aplysinella rhax* (de Laubenfels, 1954) [60] and *A. strongylata* (Bergquist, 1980) [59] showed the presence of only psammaplin and spirooxepinisoxazolines, respectively, providing many derivatives of the psammaplin and psammaplysin classes of compounds and clearly displaying high biosynthetic preference toward these over SIAs.

*Verongula* Verill, 1907 [61] demonstrated the same trends as *Pseudoceratina,* with many mSIA and bSIA compounds documented across all species. The major difference is the lack of BT and BTOx compounds reported, which suggests that this genus efficiently converts these classes into bSIABTs, mSIABTs, BTGdns or BTOxGdns.

**Figure 2:**
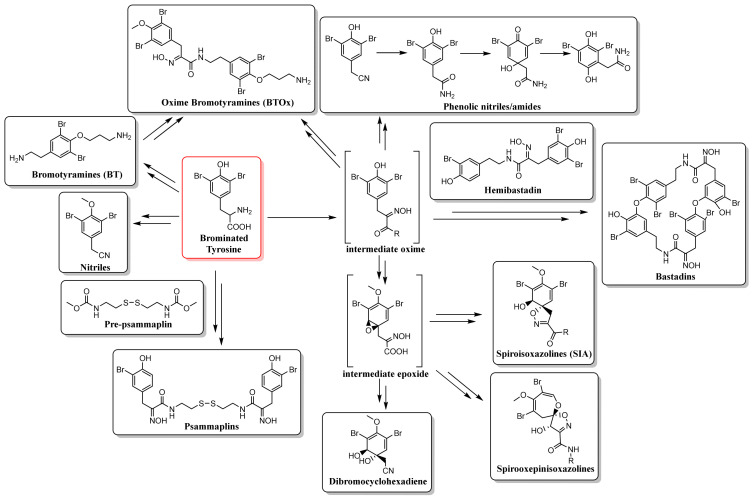
Proposed biosynthesis of BTAs from brominated tyrosine [9,11,12,64].

The biosynthesis of BTAs has yet to be completely elucidated, but it is thought to begin with the catalysed hydroxylation of phenylalanine to form tyrosine, followed by bromination through flavin-dependent halogenases. Brominated tyrosine is thought to be the source of psammaplin compounds arising through reaction with, in the case of psammaplin A, pre-psammaplin, which is derived from cysteine. Alternatively, the production of methoxylated nitriles or oxidation of the amine group can produce an oxime intermediate which provides the basis for a cascade of phenolic nitriles and amides created through decarboxylation and dehydrogenation. The oxime intermediate can also combine with bromotyramine to form, via the hemibastadin precursor, the bastadin series of compounds. Epoxidation of the oxime intermediate can also yield the SIA ring system as well as the spirooxepinisoxazolines and dibromocyclohexadienes. It has been suggested that dibromocyclohexadienes might also be generated through the degradation of SIAs to ultimately produce a highly bioactive dienone which provides part of a wound-induced chemical defence against predation [11,62,63]. In addition, the oxime intermediate can also combine with BT compounds to produce the class BTOx (Figure 2).

### 2.2. Biogeography and Hotspots for Verongiida NPs

**Figure 3:**
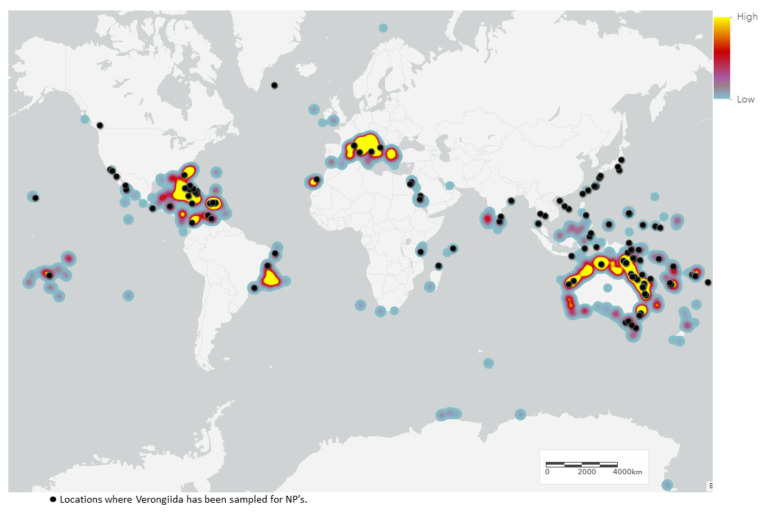
Known distribution of Verongiida sponges globally, data from Ocean Biodiversity Information System (OBIS) (heat map) [65,66]. Locations where Verongiida sponges have been sampled, yielding natural products (black dot).

Sponges of the order Verongiida are known to predominately inhabit tropical and temperate regions of the world, being present in the Central Indo-Pacific, Tropical Western Atlantic and Temperate Australasian realms (Figure 3). These sponges are found to dominate deeper reefs in the Caribbean region and New Caledonian waters, as well as the southern and eastern coasts of Australia, including the Great Barrier Reef (GBR).

Despite being relatively well-distributed throughout temperate and tropical environments, NP studies have been focused on specimens collected from the Central Indo-Pacific and Tropical Atlantic regions. This is understandable, as these regions provide exceptional biodiversity and are thus prime sampling locations for providing chemical diversity. However, this leaves several regions considerably understudied, including the western coast of Brazil (within the Tropical Atlantic realm) as well as Temperate Northern Atlantic waters, especially the Mediterranean and Adriatic Seas. These regions offer potentially untapped NP resources that should be investigated in more detail.

Regions in Japan, north of the Okinawa prefecture, have been a rich source of sponges from the genus *Pseudoceratina*, as have Hachijo-jima Island [67,68,69] and Oshima-shinsone [70], and have all yielded NPs from *Pseudoceratina* sponges, despite not being listed as regions of frequent habitation for Verongiida sponges by the OBIS. This has also been true for regions in China, such as Hainan Island [71,72] and Yong Xing Island [73], as well as Tonga [24,74] and the Gulf of Thailand, near Kho Chang Island [75]. This same trend was also observed for *Hexadella* sponge samples sourced in Jervis Inlet, British Columbia, Canada [76,77] and the *Aplysinella* sponges sampled from Pingelap Atoll, Micronesia [78]. These occurrences draw attention to the large body of biodiversity and geographical distribution data that are still undescribed for the phylum Porifera [79].

**Figure 4:**
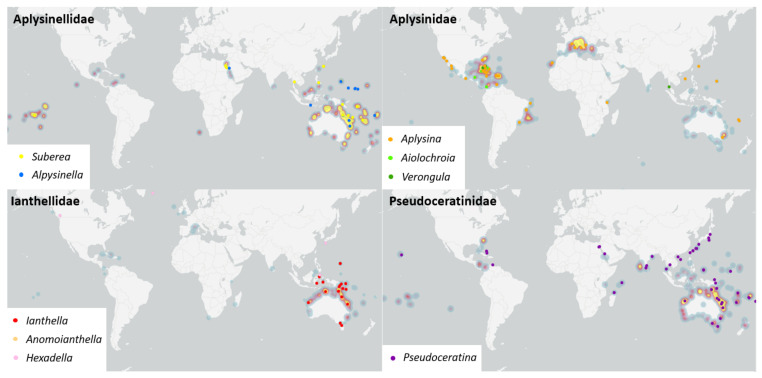
OBIS distribution of Verongiida sponge families (heat map). Locations of Verongiida sponges that have been sampled yielding NPs (coloured dots).

Variation in distribution and sampling was observed across all families of Verongiida. In some cases, geographical variation was linked to chemical differences even at the species level. While this dataset cannot allow for a full description of metabolomic differences between each species, it can still be instructive to explore the more apparent cases of metabolomic divergence observed between NPs reported in the literature. The Central-Indo Pacific realm, including the GBR, is an area that has shown the largest number of sampled and studied Verongiida sponges across nearly all genera (Figure 4). The sheer number of diverse NPs attests to this region’s biodiversity.

The genus *Pseudoceratina*, belonging to the family Pseudoceratinidae Carter, 1885 [53], has been one of the major genera sampled and studied in this region, with the most highly represented species being *P. purpurea* (Carter, 1880) [80] and *Pseudoceratina.* sp., although sampling was not limited to these species, with *P. durissima* (Carter, 1885) [53,81] and *P. verrucossa* (Bergquist, 1995) [50,82,83] also being studied from the GBR and New Caledonian regions (Figure 4). The Tropical Atlantic realm, including the Bahamas, has yielded exclusive species, including *P. crassa* Hyatt, 1875 (= *Aiolochroia crassa* Hyatt, 1875) [57,84,85,86], and no other Pseudoceratinidae species. *P. crassa* displayed the presence of several verongiabenzenoids, as well as SIAs and BT compounds, together with cyclitol glycolipids, which is unique to this species within the Verongiida order.

The Red Sea, in the Western Indo-Pacific, was another realm that showed species specificity, proving to be the only location outside of Madagascar [87] where *P. arabica* (Keller, 1889) [88] was sampled and studied for NPs [89,90,91]. This species yielded BTs, verongiabenzenoids and spirooxepinisoxazoline compounds, with some exhibiting a rare 2-(methyl)cyclopent-4-ene-1,3-dione moiety as well as incorporating BTs into their structure [91]. However, no related mSIABT compounds were reported from this species despite having the necessary BT precursors. Pseudoceratinidae sponges have been sampled and studied from many of the regions that show high levels of distribution, except for the South Pacific Ocean near the French Polynesian shelf.

Ianthellidae displays far more localised distribution, with much of the sampling occurring within the Central-Indo Pacific realm surrounding the GBR and Papua New Guinea (PNG) regions [92], although some species, such as *I. flabelliformis* (Linnaeus, 1759), can be found at more southerly latitudes, such as the Port Phillip Bay region in Victoria, Australia. Comparisons of the NPs reported for *I. flabelliformis* sampled within the southerly region of Port Phillip Bay [93], and its two North Australian counterparts in Shelburne Bay, Queensland [94] and Darwin Harbour, Northern Territory [95], yielded quite different chemistry. The Port Phillip Bay sample showed an interesting array of lactam sesquiterpenes, as well as some more common indole alkaloids, two classes unrelated to the BTA biosynthetic pathway, whereas the two northern locations displayed SIAs as well as bastadins. Bastadins represented the more common biosynthetic outcome for *Ianthella,* but SIAs proved to be a more significant find, as products of the isooxazoline biosynthetic route are far more rare from *Ianthella* sponges [96]. Macrocyclic bastadin isomers where also isolated from *I. flabelliformis* sampled from PNG [97].

The species *I. basta* (Pallas, 1766) [98] is the most studied sponge of the family Ianthellidae, and has been sampled across a number of localities, including Guam [99,100], PNG [97,101,102,103], Indonesia [104,105,106,107,108,109], GBR [110,111,112], and the Exmouth Gulf, Western Australia [113,114]. All samples across all localities yielded a very similar chemistry, proving *I. basta* to be a large producer of bastadins and their precursor, the hemi-bastadins. The only notable difference was that sponges sampled from both Guam and the Exmouth Gulf both exhibited sulfated monoesters of the bastadins and hemibastadins.

*Ianthella quadrangulata* (Bergquist, 1995) [92] was collected from three locations across the GBR. The Heron Island collection, which was the most southerly of the three, produced the most interesting chemistry [115,116]. This sample provided many bastadin congeners, but more significantly several novel dimeric brominated benzofurans were discovered, all of which were shown to have incorporated *O*-sulfate esters into their structures. This indicated that perhaps this is a biosynthetic trend that persists throughout the genus *Ianthella* and is not specific to a single species. Collections from Orpheus Island [37] and Sykes Reef [117], which were more northerly locations, appeared to mostly yield bastadin congeners, as well as an octopamine derivative. Interestingly, a number of sponges were sampled from the GBR region that were only identified at the genus level, *Ianthella.* sp. Additionally observed from this collection was a series of benzofuran compounds very similar to those produced by *I. quadrangulata* [118,119]. Unfortunately, sample details only reported GBR as the region of collection so it could not be confirmed if benzofurans are more likely to be found in Heron Island *Ianthella* sponges. Collections of *Ianthella.* sp. from the Bass Strait region of Australia yielded the most unique chemistry for the genus *Ianthella*, including pyrrolidones, lamellerins, a new class of furanones and a rare class of pyrrolidone–lamellerin hybrids called the dictyodendrins, all of which are highly unusual for *Ianthella* [120,121]. This is significant, as it appears that *Ianthella* sponges sampled from more southerly locations such as the Bass Strait and Port Phillip Bay appear to display more chemistry which is independent of the brominated tyrosine biosynthetic pathway.

Aplysinidae Carter, 1875 [122], sponges are almost as extensively studied as Pseudoceratinidae sponges. The genus *Aplysina* accounts for a great deal of this, especially in the Tropical Atlantic realm, where the biodiversity hotspots of the Bahamas, Puerto Rico and Cuba have yielded large numbers of NPs, as well as those from the Temperate Northern Atlantic within the Mediterranean region. A great deal of knowledge has been accumulated regarding the distribution of BTAs within this genus, allowing *Aplysina* to be used as an effective model in understanding the relationships between production of BTAs and biogeographical trends involving the study of depth, spatial differences, and seasonal variation on quantities of key BTAs.

The species *A. aerophoba* (Nardo, 1833) [123] and *A. cavernicola* (Vacelet, 1959) [124] are found in large quantities in the Mediterranean region and show somewhat similar chemistry, with both exhibiting SIAs as well as some characteristic pigments. Comparative studies have been performed on *A. aerophoba* and *A. cavernicola,* illustrating specific differences in the secondary metabolomes of these two species, despite both inhabiting similar regions of the Mediterranean. Differences were found between the two species in the relative concentrations of the key SIAs: aerothionin, aerophobin-2, isofistularin-3, and aplysinamisin-1, indicating these to be appropriate markers to differentiate the two species. During transplantation experiments, depth was found to play little or no role in the variability of the production of key secondary metabolites [125]. Depth was also shown to play little to no role in the chemical variability of *A. aerophoba* in a quantitative analysis of key BTAs, with the largest amount of chemical variability being explained by spatial scale between sampling sites. Interestingly, large variations were observed for sponges that were sampled in close proximity as well as those that were sampled with larger distances between sites, seeming to indicate that while proximity plays a role in secondary metabolism, there are also other factors contributing to this variability [126]. This became evident in a follow up study confirming the effect of seasonal variation on the quantitative variation of metabolites within *A. aerophoba* [127]. Seasonal and water temperature effects were also shown to be the major influences contributing to increased production of NPs for *A. cavernicola* [128]. In the case of *A. fulva* (Pallas, 1766) [98], sampled from locations in the Caribbean, USA (South Atlantic Bight, Georgia and Key Largo, Florida) as well as the Brazilian coastline, it was observed that while both locations consistently yielded SIA derivatives, only samples from the South Atlantic Bight location produced compounds such as aerophobin-1 with an imidazole functional group [21,129,130,131,132].

The genus *Verongula*, family Aplysinidae, has been sampled in many of the same Caribbean locations as *Aplysina*, as well as once from the Kho Ha Islets on the coast of Thailand. The *Verongula* sponges exhibited the same plethora of SIAs and verongiabenzenoids [133,134,135,136] that could be found across much of the rest of the order Verongiida; however, a unique array of brominated tryptamine-derived alkaloids, merosesquiterpenoids and a benzonaphthyridine were also reported. As well as being new to the family Aplysinidae, these compounds were also previously unreported within the order Verongiida, and thus have no biosynthetic precedents within this order, making *Verongula* unique amongst its Verongiida counterparts [137,138,139,140,141].

*Aplysinella* and *Suberea*, both within the family Aplysinellidae Bergquist, 1980 [59], were sampled mainly from the Central Indo-Pacific realm and accounted for the majority of NPs reported for this family. *A. rhax* was sampled from the GBR [142,143], Fiji [144], Palau [145], Guam [16,145] and Micronesia [145]. The production of psammaplin type compounds was conserved across all five locations with several derivatives of this class being reported. These studies also confirmed that despite different sample locations, the production of the pharmaceutically significant NP psammaplin A was conserved in *A. rhax*. Within this genus, *A. strongylata* was also studied and appeared to exhibit a different class of BTAs to *A. rhax*, and has yet to have any psammaplin type compounds isolated from its crude extracts. *A. strongylata* was only sampled from Tulamben beach, Bali, Indonesia, and produced large quantities of the spirooxepinisoxazoline compounds, psammaplysins [146,147,148]. Unlike *A. rhax*, which produced the disulfide psammplins, *A. strongylata* produced no BTAs that incorporated sulfur into their structure.

*Aplysinella* sp. specimens from Micronesia were reported with both spirooxepinisoxazolines and SIAs representing both the spirocycloheptadiene and spirocyclohexadiene ring structures [78,149,150,151]. This is of particular interest, as both spiro systems are thought to be biosynthetically derived from the same arene oxide intermediate [9,12]. This suggests that *Aplysinella* sponges possess the ability to produce NPs using both biosynthetic pathways. A separate *Aplysinella* sp. yielded BT compounds that were also found to be present in several *Aplysina* and *Pseudoceratina* sponges across many biogeographical realms [38,151]. Another *Aplysinella* sp. was also sampled from the Red Sea, yielding a very similar set of secondary metabolites to that of *A. strongylata*, with both producing psammaplysin derivatives showing very little biosynthetic divergence in the secondary metabolites isolated [152].

The genus *Suberea* showed similar geographic distribution to *Aplysinella* but displayed quite different chemistry. *S. ianthelliformis* (Lendenfeld, 1888) [153] sourced from the GBR [32] and Solomon Islands [34] was reported to have BTs that contained higher-molecular weight compounds than those of other genera, with many of the BTs appearing to have incorporated putrescine into their structures, reminiscent of aerothionin. However, *S. ianthelliformis* sampled from French Polynesia [154] was reported to produce unsaturated BTs as well as some quinoline derivatives. Quinoline derivatives were also identified in a study performed on a sample of *S. creba* (Bergquist, 1995) [50], sampled from the Coral Sea, but were confirmed to be produced by the isolated symbiont *Pseudomonas* sp. [9]. Curiously, other samples of *S. creba* obtained from the Coral Sea showed the presence of an array of common small molecular weight amides and nitriles such as subereaphenols, dibromoverongiaquinols and aeroplysinins, making these two *S. creba* samples quite distinct from each other [9]. The sponge *S. clavata* (Pulitzer-Finali, 1982) [155] was only sampled from the GBR and produced some species-specific clavatadines as well as some SIA derivatives, all of which incorporated Gdn as a functionality [35,36].

**Figure 5:**
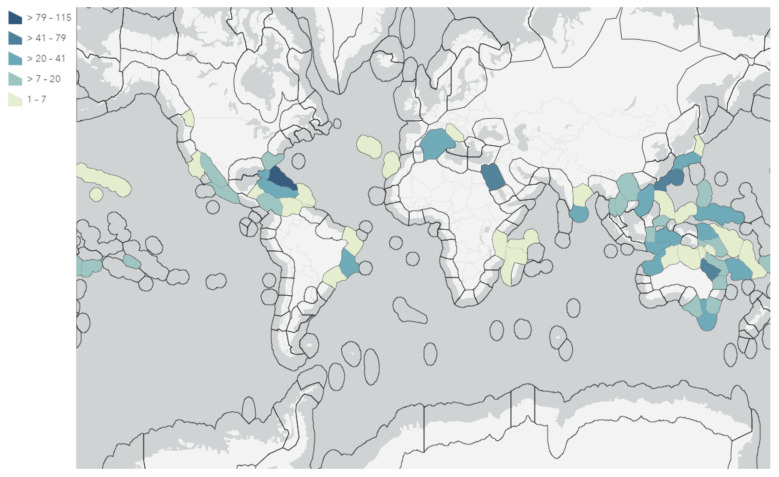
Marine ecoregions of the world (MEOWs) (Black borders) [156] displaying the total number of NPs isolated from Verongiida sponges in each region.

Marine ecoregions were used to display the frequency of isolated NPs from Verongiida sponges (Figure 5) and appear to suggest that the frequencies are highly location-dependent, with regions such as the Caribbean, GBR, the Red Sea and the Okinawan coast producing the largest numbers of NPs. Aside from the Okinawan coast, all these regions are known to have sponges that yield NPs from all four families, making them both significant Verongiida habitats as well as being diverse. However, this result may be misleading, as the regions studied are likely to have been targeted because they are readily accessible and are known to be rich sources of many tropical marine species, including sponges. Hence, the lack of study may simply reflect a lack of opportunity, or a reduced interest in other regions.

### 2.3. Natural Product Diversity across Genera of Verongiida—A Network Analysis Investigation

Many of the Verongiida species that have been studied possess NPs derived from closely related biosynthetic pathways, and this raises the following questions:

1. To what degree do these genera differ with regard to their secondary metabolites?
2. What compound classes contribute to the largest amount of variance between the genera studied?
3. Which sponges offer biosynthetic outcomes that are the most exploitable in terms of drug discovery?

**Figure 6:**
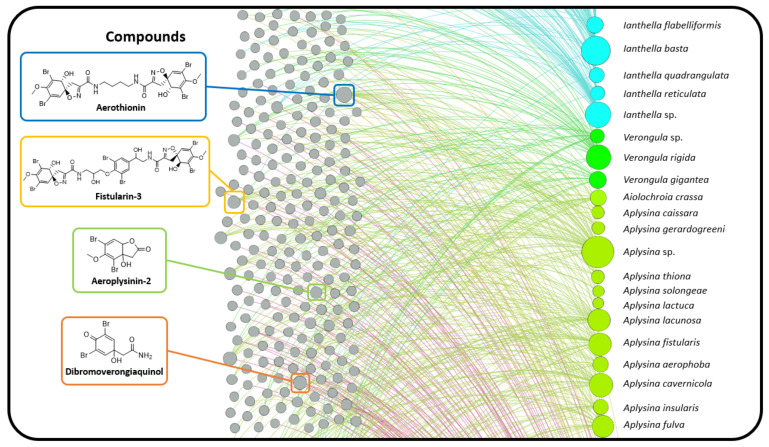
Bipartite network representation of species (nodes, right) and compounds (nodes, left) that exist within species (edges = curved lines) for the sponges in the order Verongiida.

Answers to these questions were sought using bipartite networks and chemical scaffolding methods to explore NP inter-relationships and diversity. Initially, a direct comparison of shared metabolites was created using a bipartite network consisting of two distinct classes of nodes. The first are nodes that represent every compound reported for the order Verongiida. The second type of node represents species that have had compounds reported in the literature. The network displays edges between compounds and species when a compound has been reported in the literature for that species. In this situation, no edges are created for species–species or compound–compound. To aid with analysis, node size was organised such that nodes with a higher degree (number of edge attachments) are larger than those with a lower node degree. This approach highlights the species or compounds that have the greatest interconnection, as illustrated in Figure 6**.**

**Figure 7:**
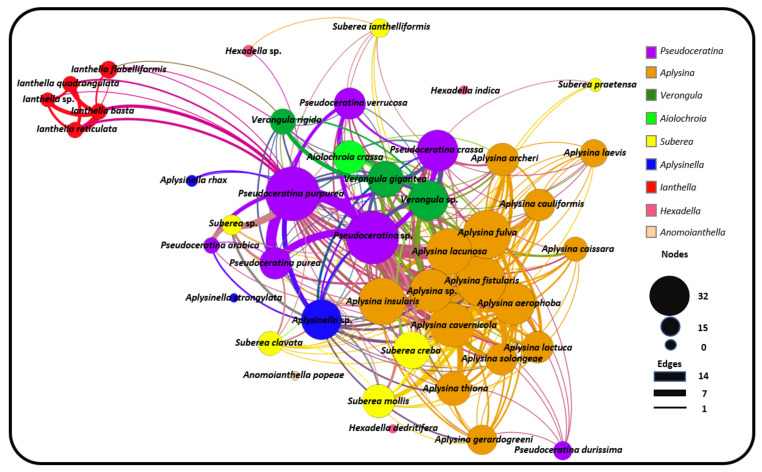
Monopartite projection of the bipartite network with respect to species (visualised using the Force Atlas layout algorithm in Gephi).

Monopartite projection of this bipartite network with respect to the species nodes gives the species network (Figure 7). In this projection, edges (curved lines) are drawn between species that share at least one compound, with a thicker edge width (weight), indicating the sharing of multiple compounds between species. The monopartite projection (Figure 7) provides a valuable visualisation, clearly indicating which species share compounds with each other. The sharing of a common compound between two species provides some evidence of shared biosynthetic pathways between the two species, thereby supporting taxonomic relationships [157]. As with the bipartite network, the node size is ordered to represent node degree, and thus indicates which species share compounds with the largest number of species within this order of sponges. Species from the genus *Aplysina* are the most interconnected in this network representation, both within the genus *Aplysina* and to external genera. This is largely due to the high numbers of common BTs and SIA compounds that these species possess, which are shared by other prominently studied species, such as the sponges from the genus *Pseudoceratina*.

**Figure 8:**
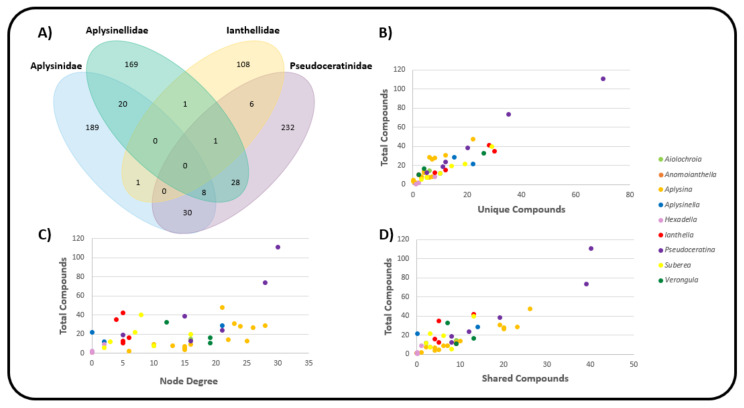
(**A**) Venn diagram for compounds distributed across families of Verongiida. (**B**) Total number of unique compounds for each species. (**C**) Node degree for each species in monopartite projection. (**D**) Number of compounds that are shared with at least one other species.

The relatively central positioning of the *Pseudoceratina* nodes illustrates the commonalities this genus displays with many other species from different families, including the *Ianthella* genus. *Ianthella* forms a unique cluster, in the large part due to the high numbers of shared bastadins that they all possess. While *Ianthella* is observed with many intra-genus edges, nearly all inter-genus linkages observed for the entire genus *Ianthella* are with the sponge species *P. purpurea*. The positional isolation of Ianthellidae sponges in Figure 7 seems to be conserved for other genera of this family, such as *Anomoianthella* Bergquist, 1980 [59] and *Hexadella*, which also exhibit very small node degrees, together with a tendency to only form edge relationships within their respective genera or not at all. Figure 8A shows the cross section of the Verongiida sponges by family, where Ianthellidae sponges are shown to have the lowest number of inter-family compounds. Interestingly, sponges of the genus *Suberea* display sharing of compounds with a higher number of inter-genera species, whilst almost no intra-genera connections are observed. This can also be seen with the genus *Aplysinella*, where *Aplysinella.* sp. shows wide ranging connections across several species outside the *Aplysinella* genus.

Initially, it was thought that the amount of interconnection amongst other species in the monopartite projection could simply be explained by the fact that species with a higher total number of compounds reported in the literature would be more likely to have a high node degree, or rather, a higher number of species with shared compounds. However, this was shown to be false, as Figure 8C shows the distribution of node degree from the monopartite graph with respect to the total number of compounds reported for each species. Except for the two *Pseudoceratina* species that have higher than usual numbers of reported compounds, there appears to be little or no correlation between the total number of compounds reported and the number of species with shared compounds. Whilst it is true that some *Aplysina* species have both high numbers of compounds and high node degrees, it can also be concluded that some *Ianthella* species have a high total number of compounds reported but a low node degree. This type of variance can also be seen within the genus *Suberea*, where both situations can be observed.

The distribution of the total compounds reported was mapped against both the number of unique compounds (Figure 8B) and the number of shared compounds (Figure 8D). In both situations, a positive correlation was observed across all Verongiida sponges. In Figure 8B,D, it is apparent that many of the *Aplysina* species exhibit far more common chemistry than the other genera in Verongiida, as they display a lower number of unique compounds per total reported compounds, as well as many shared compounds per total reported compounds. Compounds from the genera *Ianthella, Suberea* and *Aplysinella* exhibit greater uniqueness and a lower amount of relative sharing. *Pseudoceratina* shares many compounds with a large number of species, as well as having many unique compounds.

**Figure 9:**
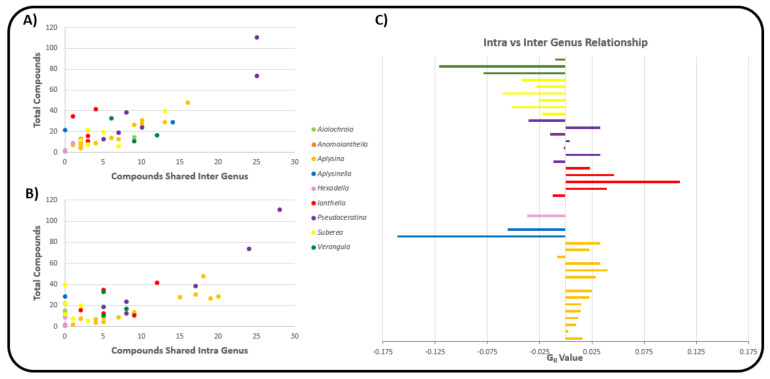
(**A**) G_R_ Value assessing overall intra- vs. inter-genera sharing of compounds. (**B**) Number of compounds shared with species of the same genus. (**C**) Number of compounds shared with species of different genera.

While Figure 8D shows a trend of shared compounds from one species to another, it is not clear if the compounds are being shared according to any predictable pattern. Keeping taxonomical distance in mind, one would expect species within the same genera to share many compounds, as they have closer genetic ties, and their biosynthetic processes are expected to be similar. As a way of investigating this ‘shared compound hypothesis’, the total compounds reported were mapped against both the intra-genera sharing of compounds and the inter-genera sharing of compounds, and the results are presented in Figure 9A,B, respectively.

The data in Figure 9A support the earlier conclusion from the monopartite projection graph, namely that the *Suberea* sponges appear to show minimal intra-genus compound sharing, and that *Aplysina* sponges display both large intra- and inter-genus sharing of compounds.

To quantify this difference between intra- vs. inter-genus sharing of compounds, the genus relationship (*G_R_*) value was calculated for each species according to Equation (1). In this instance, species are compared via their number of intra-genus shared compounds (*C_intra_*) and their number of inter-genus shared compounds (*C_inter_*). The genus relationship value was then calculated with respect to total reported compounds (*T_C_*) and the total number of species for a particular genus (*G_S_*), so comparisons are possible between species across differing genera.

(1) $$G_{R}=\frac{\left(C_{intra}-C_{inter}\right)}{T_{C}}÷G_{S}$$

The results of the *G_R_* score for each species are illustrated in Figure 9C. Values that are positive show a tendency for that species to share a larger proportion of compounds with species in the same genus. Negative values show a higher propensity to share compounds with inter-genera species. Figure 8C, derived from the monopartite projection, indicates that species within the *Aplysina* genus of sponge appeared to share compounds with many species of sponge within the order Verongiida. However, reference to Figure 9C and the associated G_R_ values of the *Aplysina* sponges indicates that much of the compound sharing for the *Aplysina* sponges occurs within the genus *Aplysina*. This suggests that *Aplysina* sponges are largely insular with regard to sharing compounds between species of sponges. However, because the *G_R_* value focusses on the relationship between intra- and inter-sharing, it misses an interesting cross section of *Aplysina* species that share the same compounds both with intra-genus species and inter-genus species. The monopartite projection (Figure 7) shows the most intra-shared compounds for each major genus, together with the compounds that are most shared between inter-genera species. In this instance, it is evident that a large proportion of the most shared compounds within the *Aplysina* genus are also shared with species from other genera, such as *Pseudoceratina* and *Suberea*.

Sponges within the genus *Ianthella* show similar trends to *Aplysina*, with largely positive *G_R_* values; however, when considering Figure 8C, *Ianthella* sponges show lower numbers of species that have shared compounds. Figure 8B also shows that *Ianthella* sponges, as a genus, exhibit many more unique compounds compared to those of the *Aplysina* sponges.

Figure 8D shows that the *Suberea* sponges have a variety of species that contain shared compounds, but only have negative G_R_ values. This is noteworthy, because while *Suberea* shows extensive inter-genus sharing of compounds, these species also have a significantly larger number of unique compounds, as is the case for the *Ianthella* species.

It has been made clear from investigating the most shared compounds from both intra- and inter-genera species that, with the possible exception of the bastadins found in *Ianthella* sponges, there is no class of compounds that is shared almost exclusively in an intra-genera manner specific to one genus and no other. Rather, there appears to be a subset of compounds that are common to all, or nearly all, species across multiple genera. There are also compounds that are specific to species in each genus, but this may also be found outside that genus. This creates uniqueness for that species within its genus, but this does not mean it is unique when considering the entire order.

Scaffold analysis was performed on compounds that were reported for each genus, with the aim of investigating the frequency of each chemical scaffold for each genera, together with assessing the diversity and novelty of the chemistry in each genera. Murcko scaffolds (N) were created from NPs (M) and used to calculate genera diversity (N/M), where the frequency of each Murcko scaffold indicated importance to the genera. Scaffolds that existed in only one genus were termed scaffold singletons (N_sing_) and used to calculate genera novelty (N_sing_/M) (Table 2) [158,159].

**Figure 10:**
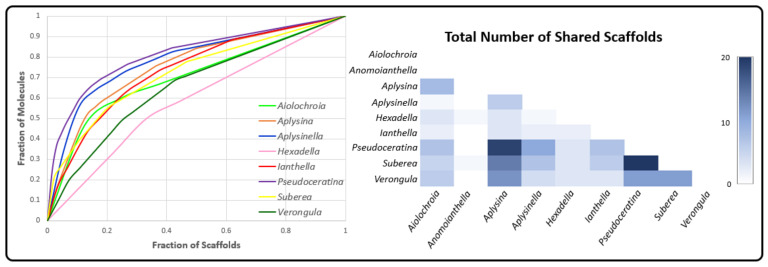
Cumulative scaffold frequency by genus (**left**). Number of shared scaffolds by genus (**right**).

A high diversity of scaffolds was reported for *Verongula* and *Suberea*, with diversity scores of 0.549 and 0.409, respectively. These were significantly higher than other genera such as *Pseudoceratina* and *Aplysina*, which appear to have a larger number of compounds that are represented by a relatively low number of scaffold classes. Furthermore, they also displayed low novelty scores and there were many shared scaffolds between the two genera (Figure 10). While novelty and diversity are advantageous for drug discovery efforts, a lack of these properties combined with many shared scaffolds could suggest similarities in terms of the biosynthetic origins of the compounds produced by two genera, thereby also providing chemotaxonomic value. It should be noted that although *Aiolochroia* Wiedenmayer, 1977 [160], *Anomoianthella* and *Hexadella* also achieved much higher diversity scores as well, it is likely that this is simply a result of the low number of NPs reported in the literature for these genera. Cumulative scaffold frequency graphs of each genus show *Pseudoceratina* to have the highest number of compounds represented by the lowest number of scaffolds. Interestingly, the genus *Aplysinella* shows a similar trend, with a sharp rise at the beginning of the curve indicating an upper end of scaffolds that dominate its dataset.

**Figure 11:**
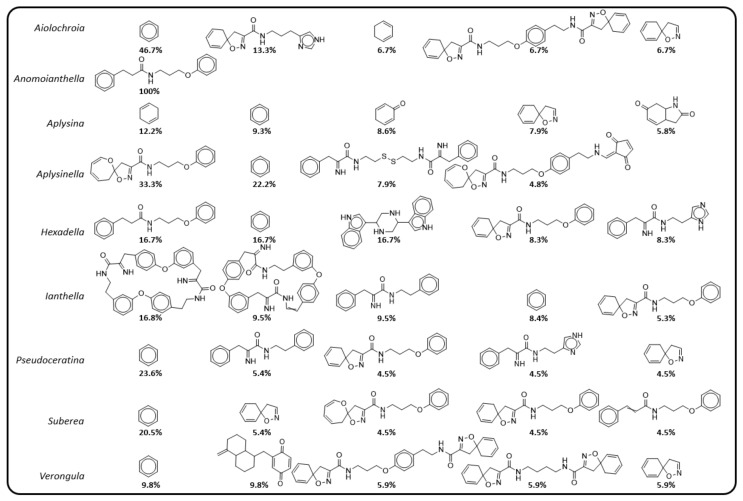
Most frequently occurring Murcko scaffolds for each genus.

*Pseudoceratina* has approximately 23.6% of its compounds represented by the benzene Murcko scaffold (representative of phenolic nitriles, amides and BTs), as well as SIA (4.5%) and BTOx (5.4% and 4.5%) scaffolds (Figure 11). *Aplysinella*, on the other hand, has many more scaffolds created through the alternate biosynthetic route of spirooxepinisoxazolines. *Hexadella* and *Verongula* both display curves representing a low ratio of molecules per scaffolds, indicating the presence of only a small number of derivatives present in their listed NPs. This type of data analysis can inform future isolations and could provide evidence of two situations: (i) that the organism only produces single derivatives of the same scaffold, or (ii) that there simply has not been many of these derivatives discovered. Either way, the combination of high scaffold diversity and novelty that *Verongula* displays, together with the low ratio of molecules to scaffolds, make this genus an ideal target for drug discovery, with the potential to provide novel scaffolds as well as derivatives of known scaffolds.

The number of shared scaffolds between *Pseudoceratina* and *Aplysina*, the two genera with the highest number of reported NPs, implies a strong biosynthetic connection. *Aplysina* and *Pseudoceratina* have many compounds represented by the SIA scaffold with 7.9% and 4.5% for each, respectively. This, together with the high frequency of benzene, demonstrates the utilisation of a number of common biosynthetic routes to achieve these scaffolds. However, *Aplysina* appears to produce more compounds that have lower MW Murcko scaffolds such as BTs, cavernicolins, bromotyrosineketals and verongiaquinols, whereas *Pseudoceratina* has higher MW scaffolds that are created from the NP classes of BTOx, SIABTs or BTOxGdns. *Suberea* sponges exhibit a variety of scaffolds from *Aplysinella*, *Pseudoceratina* and *Aplysina,* with a mixture of SIAs and spirooxepinisoxazolines. This subset of common scaffolds, which are shared between the more widely studied genera within the Verongiida order, suggests that all biosynthetic outcomes of the BTA class of compounds are available to each genus, but variance is created at the species level. Considering this, it would be useful to understand if species share more in common with their intra-genus counterparts than with inter-genera species.

**Figure 12:**
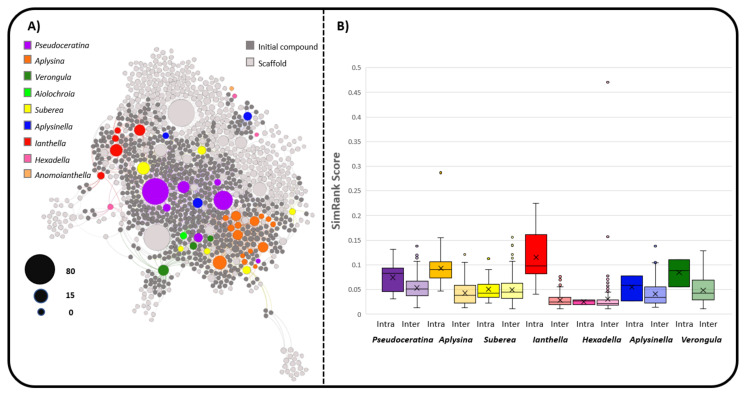
(**A**) Scaffold network (SN) created using HierS type scaffolds that displays species (coloured), initialised compounds (dark grey) and scaffolds (light grey). (**B**) SimRank score data calculated for each species comparison in the SN, organised via genera comparing intra/inter genera relationships.

To this end, scaffold trees were created using the RDKit scaffolding package [161] and were arranged to create a scaffold network (SN) as presented in Figure 12A. These networks include nodes representing individual species, whole compounds (initialised compounds), as well as common scaffolds created from whole compounds. Edges (links) between species nodes (coloured) and initialised compounds (dark grey) represent a compound that has been reported for that species. Each compound is iteratively fragmented into its substituent scaffolds, and edges (links) are placed between the initialised compounds and the scaffolds (light grey). This provides a chemical space where species are placed based on the structural features of their associated secondary metabolites. Scaffolds exist between two species that would have otherwise not been connected in a bipartite network projection due to them not having a single metabolite in common. As species are distributed according to their metabolites, it is possible to assess their likeness to each other with respect to their local environments within the network. Environmental similarity was assessed using the python networking tool SimRank [162], where a domain-specific view of each species was taken. In this way, species were compared to each other based on their respective environments where two objects are similar to each other if they are both connected to similar objects.

Figure 12B presents the box plot results of SimRank scores between all species nodes in the SN created by considering intra-genera versus inter-genera similarity comparisons. SimRank scores show similar trends to the G_R_ score method of comparison when considering *Ianthella* and *Aplysina*, where there is a strong tendency towards similar chemistry within their respective genera, as opposed to species from other genera. This trend is due to the large number of unique bastadin compounds that are found in the genus *Ianthella,* and the high density of common SIA compounds that are found in *Aplysina,* which are extensively shared within the genus, whilst also exhibiting very similar scaffolding.

*Suberea* show very low numbers of shared compounds within the genus, but it appears that there is a very similar number of common scaffolds both intra- and inter- genera when considering the SimRank results for this genus. *Suberea* shares a large proportion of SIAs with other genera, such as *Aplysina* and *Pseudoceratina,* but also displays many compounds that are species-specific to *Suberea* and have similar scaffolds and overall chemical structures.

The genera *Aplysinella* and *Verongula* appear to have more inter-genera shared compounds when considering G_R_ score, but when considering chemical features and common scaffolds in the SN, there is a larger proportion of similar scaffolds in their intra-genera species as opposed to inter-genera species. This is likely to be reflective of the fact that the G_R_ score, while useful for direct comparisons of shared compounds across species, does not accurately depict the relative chemistry of species that have only very low numbers of connections in the monopartite projection of shared compounds. If a species is present with only a very low number of shared compounds for both inter- and intra-genus comparison, there is little that can be concluded without further information regarding the chemical classes present. *A. rhax* and *A. strongylata* have very limited interconnection with both inter- and intra-genera species (Figure 7), leaving the trend for the genus *Aplysinella* to be dictated entirely by *Aplysinella.* sp. This results in a misconception when comparing genera via shared compounds, because it is entirely likely that species within a genus will produce compounds that are structural variants and thus will not be reflected in sharing, but rather in shared or common scaffolds. This means that while shared compounds can provide a useful insight into shared biosynthesis, it needs to be considered together with shared common scaffolds to provide context to the compounds that are not shared frequently, and the compounds that are potentially missing from the data. This is most important, as not all datasets are complete, especially in NPs research, where datasets are subject to how rigorously each species has been investigated and by what methodologies.

### 2.4. Verongiida NP Drug Score and Drug-Likeness Assessment

**Figure 13:**
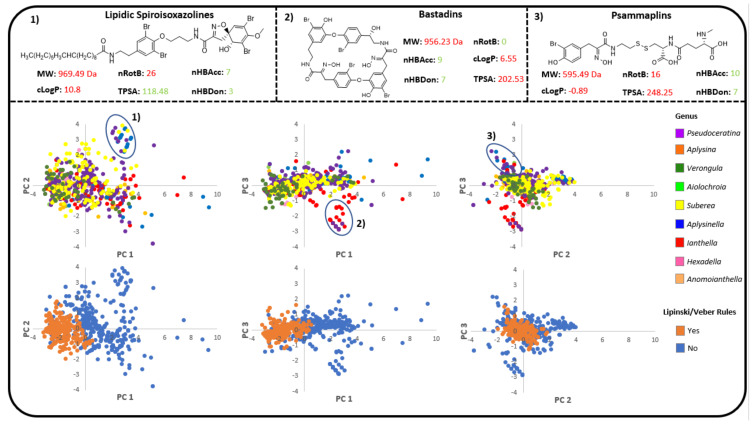
PCA analysis of chemical descriptors MW, TPSA, nRotB, nHBDon, nHBAcc and cLogP.

The compounds produced by Verongiida sponges were assessed for their pharmaceutical potential using both the Lipinski/Veber rules and the drug score metric calculated on the OSIRIS property explorer [163]. Chemical space was first represented using principal component analysis (PCA) with chemical descriptors derived from the Lipinski/Veber rules, such as molecular weight (MW), topological polar surface area (TPSA), number of rotatable bonds (nRotB), total number of hydrogen bond donors and acceptors (nHBDon/Acc) and the octanol water partition coefficient (cLogP), forming the basis of compound features, with the results illustrated in Figure 13. Descriptive statistics of the PCA plots in Table 3 show that 93.5% of the cumulative variance of this data is described by the first three principal components. Table 4 shows that PC1 has a strong positive correlation with the descriptors MW, TPSA, nHBDon as well as nHBAcc; PC2 has a large positive correlation with the cLogP coefficient and nRotB; while PC3, which contributes to only 8.4% of variance, has a positive correlation with the nRotB descriptor and a negative correlation with cLogP.

Most outliers in these PCA plots are accounted for by variance in PC1, with a few associated with variance in PC2. Some small cluster groupings can be observed where compounds from *Suberea*, *Pseudoceratina* and *Aplysinella* form a cluster of spiroisoxazoline compounds that have long lipidic tails. This cluster is formed due to the compounds displaying unusually large cLogP and nRotB values, which corresponds to the two descriptors showing large positive correlations with PC2 in Table 4. Some clustering was also observed along the PC3 axes, which can be accounted for by the bastadin compounds, which are found primarily in the genera *Pseudoceratina* and *Ianthella* and are unique, as they are the only macrocyclic compounds found in this order of sponge. This often results in high MW compounds that have very low numbers of nRotB. The psammaplins were also observed to form a small cluster when comparing PC2 and PC3. These compounds exhibit the only disulfide functionality across the entire order, and are found in *Aplysinella*, *Ianthella* and *Pseudoceratina* sponges. Outliers in this analysis were found to be highly lipophilic compounds that exhibited large cLogP values, MW and TPSA values.

**Figure 14:**
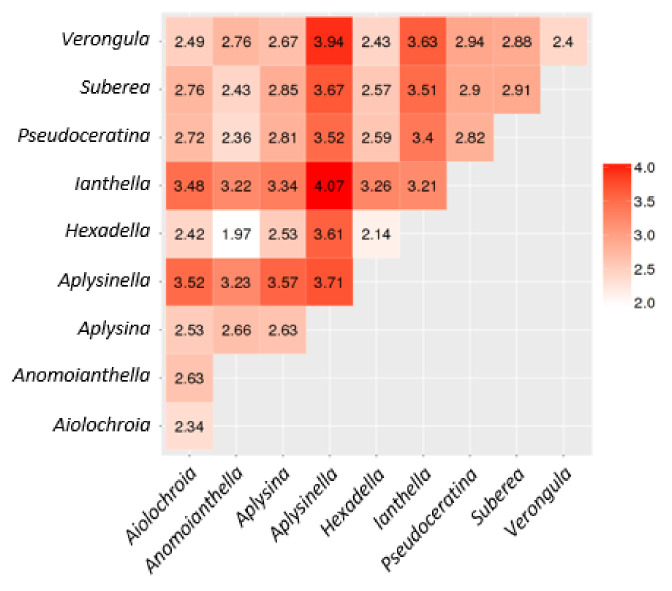
Euclidean distance of genera based on pharmacokinetic properties of compounds.

The Euclidean distance between each genus, which correlates with their degree of relatedness, was studied and the results are summarised in Figure 14. Euclidean distance measurements were performed using the PUMA cheminformatics server [164,165]. This measurement of similarity between genera appeared to display similar trends to the SimRank score plot, despite using pharmacokinetic features to describe molecular structure rather than chemical scaffolds. 

The genus *Ianthella* showed a smaller Euclidean distance of 3.21 when compared to itself, whereas the inter-genus scores against all other genera were found to be larger in magnitude. *Aplysina* had a short Euclidean distance value of 2.63; however, it showed smaller distances when compared to *Aiolochroia* and *Hexadella,* which both scored distances of 2.53. *Pseudoceratina* appeared to have higher inter-genus distances, compared to the intra-genus value of 2.82, than *Suberea*, *Verongula* and *Aplysinella*, which showed values of 2.9, 2.94 and 3.52, respectively. 

This contrasted with the values observed with other genera, which were all much lower. *Ianthella*, *Aplysinella* and *Verongula* are suggested to have the most unique chemistry based on the descriptors, and these provided the highest Euclidean distances. This tends to agree with the SN results and the PCA plots. *Aplysinella* shows the greatest amount of variance along PC2, due primarily to its compounds that have a large range of cLogP values. It also has a single species in the SN that displays highly unique scaffolding and a very large distance to other genera. The entire genus *Ianthella* shows unique scaffolding in the SN and also relatively large data variance across all three principal components, as shown in Table 3. *Verongula* appears to derive most of its uniqueness from the variance along principal component 1 and the fact that, as with *Aplysinella*, it has a single species that produces unique scaffolds in the SN.

PCA analysis showed large numbers of compounds from *Pseudoceratina*, *Verongula*, *Suberea*, *Aplysina* and *Aplysinella* occupying the chemical space of Lipinski/Veber-abiding compounds. Many of the compounds that do not abide by these rules were discounted due to excessively high MW, cLogP and/or nRotB values.

**Figure 15:**
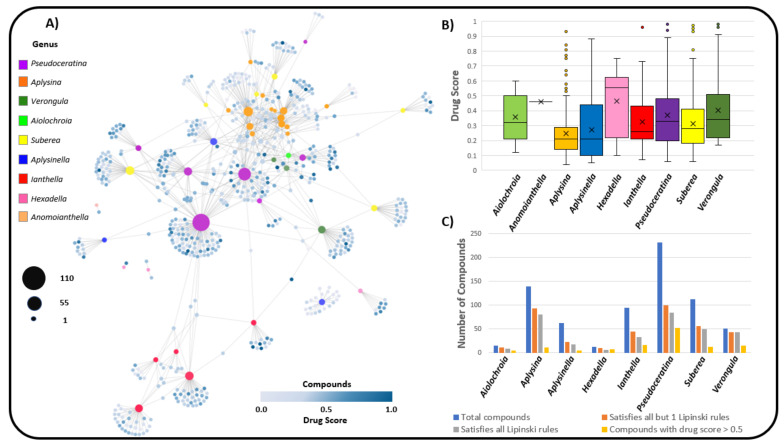
(**A**) Species drug score network. (**B**) Drug score distribution by genus. (**C**) Lipinski statistics by genus.

Figure 15A shows a network where the distribution of compounds amongst genera can be observed as well as the compound’s associated drug score, as calculated by the OSIRIS property explorer [163]. The drug score of a compound is used to assess its potential pharmaceutical value, based on parameters such as cLogP, solubility, molecular weight, drug likeness and any associated toxicity risks, on a scale between 0 and 1 (where 1 indicates a high potential to qualify as a drug). The drug score is a powerful value that encapsulates core druglike descriptors and is particularly useful for providing a snapshot of the compound’s drug feasibility, whilst also considering its predicted toxicity.

The genera that exhibit the highest mean drug score for compounds are *Hexadella*, *Verongula*, *Pseudoceratina* and *Aiolochroia*. The genera *Aplysina* has a relatively low drug score profile for its compounds, as seen in Figure 15B, but it does display a number of compounds with particularly high drug scores as outliers. In Figure 15A, it can be observed that many of the high drug score compounds for *Aplysina* are found only within *Aplysina* sponges. This contrasts with *Pseudoceratina,* where many of the high druglike compounds are found to be shared amongst other genera such as *Aplysinella,* together with many high drug score compounds that are exclusive to *Pseudoceratina*. Many other genera, especially *Ianthella*, show the same trend as *Aplysina,* where the high drug score compounds are not shared with inter-genus species. Despite having many compounds reported for both *Aplysina* and *Pseudoceratina,* of which a relatively large percentage conform to all Lipinski and Veber’s rules for druglikeness, (57.5% and 36.2%, respectively), Figure 15C shows that an incredibly small percentage of these compounds possess appropriate properties to achieve a drug score higher than 0.5, and this is attributed to the predicted toxicity and mutagenic properties of these compounds being too high to allow for an effective drug score.

**Figure 16:**
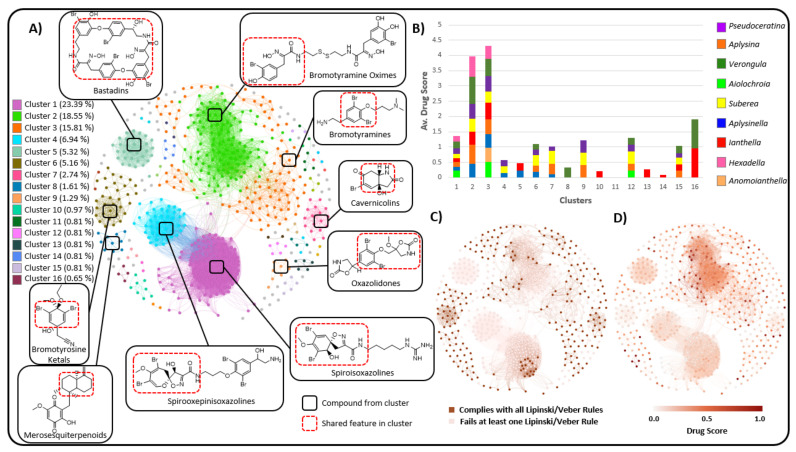
(**A**) Similarity network with Louvain clustering applied. (**B**) Average drug score for each cluster arranged by genera. (**C**) Similarity network highlighting compounds that conform to the Lipinski/Veber rules. (**D**) Similarity network ranking compounds based on their drug score.

To further explore the relationship between drug score and chemotype in this order of sponges, chemical space networks (CSNs) [166] were used to create a chemical space for compounds based on chemical similarity, as illustrated in Figure 16. Network analysis, based on chemical similarity, provides a direct connection of cluster analysis with chemotype without loss of information due to dimensionality reduction, as would be observed in PCA.

This type of network connects compounds (nodes) to other compounds based on structural similarity as expressed via the Tanimoto score between two compounds. A network threshold value of 0.5 was used to maximise the assortativity degree and average clustering coefficient, whilst also minimising the number of singletons and providing an appropriate network density. The Louvain clustering algorithm (default Gephi clustering algorithm) was used to cluster the compounds in this network. This resulted in 20 clusters having three or more nodes. Major clusters represent the major chemotypes present in this order of sponge, as can be seen in Figure 16A. Chemical assessment of the major clusters in this network showed that the predominant chemotypes present are SIAs (Cluster 1), BTOx (Cluster 2), BT’s (Cluster 3), spirooxepinisoxazolines (Cluster 4), bastadins (Cluster 5), bromotyrasineketals and verongiaquinols (Cluster 6), as well as cavernicolins and bromotyrosine lactone derivatives (Cluster 7).

The Lipinski/Veber rules and drug score ranking were then applied to this network, as illustrated in Figure 16C,D, respectively. Figure 16C shows darker nodes for compounds that comply with all the Lipinski/Veber rules. It should be noted that when comparing Figure 16C,D, compliance with the Lipinski/Veber rules does not necessarily guarantee a high drug score. This is likely due to the compounds having lower druglikeness values and/or also having high predicted toxicity values for either mutagenic or irritant properties. Upon comparing Figure 16D with Figure 16A, it was observed that the cluster of compound classes that achieve the highest drug score ranking includes the BTOx compounds (Cluster 2) and the simple BT compounds (Cluster 3). These two classes of compounds are found widely across the order Verongiida and contribute to the mean drug score of most genera.

For example, *Verongula* gains much of its high mean drug score in Figure 16B due to the presence of high drug score compounds from cluster 2, achieving a mean drug score from this cluster of 0.89, and from cluster 16, where compounds from this genus achieve a mean drug score of 0.94. For genera such as *Ianthella,* which achieve a relatively low mean drug score of 0.33 for its compounds, it can be inferred from Figure 16D that much of this can be attributed to the high prevalence of bastadin-type compounds. The macrocyclic bastadins exhibit high molecular weights, large numbers of nHBAcc and high cLogP values, all contributing to low drug scores for the *Ianthella* genera set of compounds. While these compounds lower the mean drug score for *Ianthella,* there are other clusters, such as cluster 16, where *Ianthella* shows a considerably higher mean drug score of 0.96 (see Figure 16B). This seems to suggest that *Ianthella* sponges do produce compounds with a drug potential that is higher than that of their most frequently occurring compound class, which are the bastadins (cluster 5). Information such as this is important when considering future isolation strategies and investigations for these species of sponges, as it would be wise to use an approach that avoids the isolation of bastadins, and that preferentially aims to isolate compounds such as those found in clusters achieving high mean drug scores, such as components in clusters 2 (BTOx), 3 (BT) and 16 (Aplysinopsins). This does not mean that the isolation of bastadins should be ignored, since as macrolides they may possess useful membrane disrupting properties, but rather that they should not be the primary targets for small molecule isolation.

This type of network analysis could conceivably be utilised to prioritise isolation strategies for compounds with high drug scores, although it would not provide insight into predicting which organisms would produce novel chemicals that are highly bioactive. Nevertheless, it may still be of use in designing isolation strategies that target specific chemotypes that are assessed as being likely drug candidates with higher drug scores. This type of structure-based similarity network that relies on thresholds is useful for identifying the major types of compounds present across a dataset and can provide insight into structure–activity relationships in compounds. Compounds can be compared to each other within a single network with a threshold value, but it is relatively difficult to compare those across multiple networks due to different network statistics. This, however, does not invalidate this type of network analysis in terms of understanding chemotype–drug viability relationships across chemical space.

Recent studies of BTA compounds have shown this class of compounds as being promising candidates within the preclinical pipeline [25,26,27,28,29,30,31,32,33,34,35,36,37,38]. Despite this, the large majority of BTAs from Verongiida sponges have been understudied when it comes to biological activity and function, leaving a potentially untapped resource for drug development.

**Figure 17:**
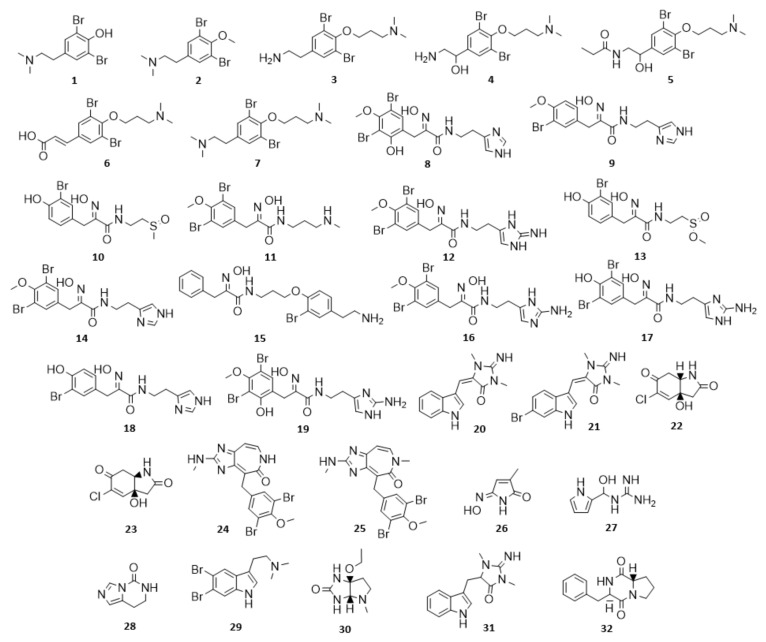
Compounds from Verongiida sponges that achieve a drug score of ≥0.75.

Figure 17 shows the compounds that have achieved a drug score of 0.75 or higher, with all molecular descriptors displayed in Table 5. Many of the compounds that achieve high drug scores are from clusters 2 (BTOx) and 3 (BT) of the CSN. In addition, a large proportion of these compounds are from miscellaneous clusters that are either singletons or simply have three nodes or fewer contributing to their cluster information. This suggests that this type of network analysis is useful for observing activity trends only when the dataset contains large numbers of compounds with high similarity, as can be observed in clusters 2 and 3.

Table 5 presents molecular descriptors for compounds that achieve a drug score higher than 0.75. Aplysamine-1 (**7**) was shown to be a weak H_3_ receptor antagonist compared to a standard of conessine achieving an IC_50_ value of 0.34 μg/mL (0.83 μM) [167]. Compounds **24** and **25** (ceratamine A and B, respectively) are cytotoxic and antimitotic in a variety of assays covering a number of human and rat cell lines [168,169,170]. Compound **28** was assessed as being a potent antifungal agent against the fungus *Geotrichum candidum* [171]. Compound **29** showed some antidepressant activity during a rodent forced swim test [138,172]. Compound **32** has been reported to be antibacterial, antimycobacterial and an effective inhibitor of human ETA receptors as well as neuropeptide Y1 receptors [173,174,175]. It is noteworthy that none of the other listed compounds have had any form of bioassay undertaken despite being ideal drug candidates that achieve high drug scores and generally conform to the Lipinski/Veber rules. This clearly presents an opportunity for further work to evaluate the therapeutic potential of these compounds.

## 3. Methodology

### 3.1. Collection of Chemical Compound Data

All compound data were manually curated from the literature and ‘data-mined’ from the SciFinder database using keyword search phrases. Keyword searches were performed on all genera that make up the order Verongiida. This process was assisted by using reviews that focus on specific genera within this order [9,10]. Curation of the literature data resulted in a library of 633 NPs that were reported from species within the order Verongiida. This represents a comprehensive list of all secondary metabolites isolated from Verongiida sponges within the period from 1960 to May 2020. It is important to note that since May 2020 to August 2021, a further 7 papers have been published concerning secondary metabolites from this order that were not included in this analysis [176,177,178,179,180,181,182]. A full reference list can be found in Supporting Information S1.

This library contains compounds isolated from 43 separate species from across 9 different genera. Of the 5 families that are contained within the order Verongiida, compounds were reported from only 4 families (Aplysinellidae, Aplysinidae, Ianthellidae and Pseudoceratinidae), as listed in Table 6.

### 3.2. Network Considerations

Graphical presentations, or networks, are a useful tool when representing chemical space that can otherwise seem inaccessible due to the sheer number of organic molecules that exist within a dataset. This is especially true when considering the number of NPs that have been documented. Networks are created by relationships observed between pairs of data points. Vertices or nodes (V) are connected by edges (E) which can be expressed by the relationship, G(Graph) = (V, E). Network distribution and topology can be defined by several metrics, including degree, density, assortativity, modularity and clustering coefficient (see Supporting Information S2).

Edges can represent either a one-way or two-way relationship between nodes, termed either directed or undirected. Undirected networks display edges that are bidirectional, meaning that the relationship between two nodes is equal in both directions. On the other hand, a directed network displays only connections that exist in one direction. In this study, the NP similarity data were calculated using the Tanimoto value which considers the global similarity of the compound structures; hence, the networks in this study were made with undirected edges.

Edges, whilst representing a relationship between nodes, can also have an associated value or weight. Networks that incorporate edge weights are termed weighted networks. In this situation, a nominal value dictates the significance of the relationship between two nodes, thereby creating certain node relationships that are more significant than others within the structure of a network. Furthermore, some networks can be both weighted and directed. In this instance, networks display edges as arrows rather than lines and will often display edge weight visually by increasing the physical thickness of edges in a network that have higher weightings. All networks in this study were undirected, with some relying on weighting values (monopartite projection network) and others being unweighted (scaffold network).

Networks can also be defined as being either bipartite or monopartite. Monopartite graphs are usually created by considering data that are of a similar kind to be represented by nodes. The network is created to investigate the relationships of objects, as is the case of graphs designed around investigating academic citation patterns, where nodes are the academics and the edges represent a single citation of one author by a second author [183]. In this situation, all nodes are the same type of data (academic authors). However, nodes within networks do not always have to be the same type of object, such as in the case when networking host–microbiota relationships in nature [184]. In this situation, it can be appropriate to use bipartite networks where two distinct groups of nodes are identified (predators and prey) and relationships, or edges, are defined between the two groups but not within each group. The work described in this current study presents the exploration of both types of networks within the scope of chemotaxonomy and in the assessment of drug viability.

Network layout is another important factor when considering how to visualise a network, as it can often have a significant influence over the utility and interpretation of the created network. The layout of a network refers to the relative topology of the nodes that comprise the network. Many layout designs exist, but the Fruchtermann Reingold algorithm [185] is the most widely used in networking chemical space, with the force atlas algorithm offered by the Gephi software [186] also being a popular choice, both of which have been used in this study.

### 3.3. Molecular Fingerprints, Similarity and Scaffolding

Chemical similarity, as a concept, is relative and highly dependent on the methods used to assign it. Considerations need to be made regarding how to view molecules when comparing them, whether it be using global molecular topology (the molecule as a whole) or whether sub-structure methodologies are employed to provide a more focused outlook on important structural motifs (see Supporting Information S3).

Similarity is calculated by considering the features of molecules and comparing the common features that two compounds share. A common method used to ascribe similarity to molecules is the Tanimoto coefficient, sometimes referred to as the Jaccard index. For molecules *A* and *B*, let *T_c_* equal the Tanimoto coefficient, where the common features of both *A* and *B* are divided by the total number of remaining features for both molecules, as defined by Equation (2).

(2) $$T_{c}\left(A,B\right)=\frac{\left|A\cap B\right|}{\left|A\cup B\right|}$$

Features that are compared by the Tanimoto coefficient are usually binary bit vectors called structural keys. The structure key used in this study was the Morgan fingerprint from the RDKit package, which is similar to the more common extended connectivity fingerprint (ECFP).

The optimisation of networks requires the use of appropriate ‘threshold parameters’ to prepare a network that achieves desirable aesthetic qualities without missing key information (see Supporting Information S4).

### 3.4. Creation and Visualisation of Networks as Applied to Data for the Verongiida Sponge Order

#### 3.4.1. Bipartite Networks

Bipartite networks were created from two different types of nodes: (i) NPs in the form of Simplified Molecular Line-Entry System (SMILES) codes and (ii) species from the order Verongiida. This network was created from the n x m matrix between all compounds and all species. The matrix entries are either 0, where a compound is not found in that species, or 1, where that compound has been reported.

The monopartite projections of this network show the relationship between species based on their shared compounds. In these monopartite projections, two species are joined by an edge if they each share a compound in the original bipartite network. Monopartite projections of bipartite networks also exhibit edges that are weighted based on the number of shared compounds between species. That is to say, the more compounds two species share together, the thicker the edge will be in a monopartite projection. Bipartite graphs and monopartite projections were created using the networkx library in Python. These were subsequently visualised using the Gephi networking software package version 0.9.2 (Supporting Information S5).

#### 3.4.2. Scaffold Networks (SNs)

SNs are undirected non-weighted networks that consist of three distinct types of node: (i) species nodes, which are nodes that are exclusively linked to initialising compound nodes (only the compounds that are reported in the literature to be found in those species); (ii) initialising compound nodes, which represent the full structure of a compound found within one or many species and must be linked to at least one species node directly, but are also linked to at least one scaffold node or possibly many; (iii) scaffold nodes, which represent the scaffolds derived from the initialising compounds. The scaffolding of compounds was performed using the RDKit scaffolds package, which follows the HierS method of scaffolding [161,187]. The subsequent SNs were prepared using in-house python applications and visualised using Gephi ver 0.9.2.

#### 3.4.3. Chemical Similarity Networks (CSNs)

**Figure 18:**
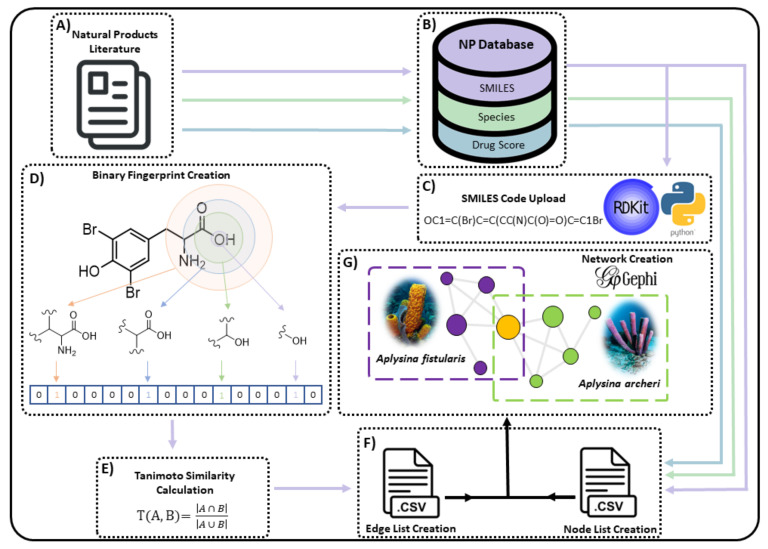
Conceptual scheme for networking NP compound libraries. (**A**) Collecting the NP literature obtained from SciFinder database with an emphasis on keyword searches such as genus and species name. (**B**) Curating the literature to form a database including all pertinent attributes of compounds such as species and sample location. (**C**) Reading and canonising SMILES codes from the NP database using in house python code and RDKit library. (**D**) Creation of a unique binary fingerprint for all molecules. (**E**) Calculating the Tanimoto similarity value for all possible compound comparisons. (**F**) Combining similarity calculations into an edge list describing all edge information for networks and creation of a node list including all nodes that will feature in the network and their attribute information from the NP database. (**G**) Using the Gephi software package to input node and edge list information to visualise the network.

All compound similarity values that were used to create networks were calculated on the basis of the Tanimoto coefficient and prepared in a similar fashion to the CSNs created by Maggiora and Bajorath [166]. Compound similarity was calculated using the Morgan fingerprint derived from the RDKit library in python. The compound library that was curated from the literature was processed prior to fingerprint creation using in-house python code to create both node and edge lists that displayed all associated attributes of each compound. Network statistics were calculated using the networkx library in python. The data curation and network creation process are summarised by the schematic in Figure 18.

### 3.5. PCA and Drug Score Assessment

Principal component analysis (PCA) was performed on compounds for each dataset using common chemical descriptors (Lipinski/Veber parameters) that include: molecular weight (MW), topological polar surface area (TPSA), number of rotatable bonds (nRotB), total number of hydrogen bond donors and acceptors (nHBDon/Acc) and the octanol water partition coefficient (cLogP) (Supporting Information S6). This, together with Euclidean distance calculations, was performed using the Platform for Unified Molecular Analysis (PUMA) and Minitab version 19.2 [164,165]. Druglikeness and the drug score for all compounds were assessed using the OSIRIS Property explorer [163]. The drug score is a value that incorporates the pharmacokinetic properties of each compound together with predicted druglikeness and the associated toxicity risk for each compound.

### 3.6. Limitations

Once the collection and collation of the scientific literature related to this current study were completed, some bias trends were noted in studies related to the extraction and isolation of NPs. Whilst the recognition in these bias trends means that improvements are being noted in more recent natural products publications, there is a need for these biases to be further addressed in order to improve the validity of cheminformatic work seeking to explore biogeographical NP trends as well as for further exploring secondary metabolite distribution amongst specific taxa. The biggest issue concerning some of these publications is related the missing or lacking taxonomic identification and geographical sampling information. This problem could be addressed with the inclusion of appropriate DNA identification of organisms for future NP studies, a suggestion made previously amongst the chemosystematics community [188]. Given this limitation, it is possible that some of the Verongiida sponge taxonomic classifications used in this current study may have been incorrectly classified in the literature, or that they have recently been re-classified as other species. Taxonomic reclassifications can occur many years after the initial studies and many of these reclassifications have been noted herein. In this current study, the taxonomic information obtained from the literature was taken on face value and in good faith. The authors have not attempted to authenticate or to challenge the taxonomic assignments reported in the research, as such a task was beyond the remit of the present work. Such an undertaking would also not be straightforward, as many publications give little, or no, details of how the taxonomy of the sponge was assigned.

Furthermore, many of the trends observed in this work may be subject to researcher bias such as geographical trends, which may simply highlight the focus of researchers on a certain area or species. For example, many data exist for the two genera *Aplysina* and *Pseudoceratina*, more so than any other genus, which is primarily due to the focus of specific NP research groups during the past 20 years. These sponges were also sampled from specific areas in the Caribbean and the Indo-Pacific, resulting in many NPs being reported from these genera and geographic locations, once again reflecting the bias of research focus. 

This dataset is also subject to the bias inherent in the extraction and isolation procedures used by the respective research groups investigating each species. The extraction solvent plays a large role in the pool of secondary metabolites that become available for investigation, providing bias across studies as well as in experimental design. Here, the focus on the isolation strategy can affect the outcome of secondary metabolites identified. This is also apparent when comparing the general strategies of isolation seeking a chemical novelty, with targeted approaches seeking a specific pharmaceutically relevant chemotype using for instance, bioassay guided fractionation. Each method of extraction and isolation can potentially miss important secondary metabolites that could be used for cheminformatic style studies.

Data analysis provided its own set of limiting factors on top of data collection which centred around the reduction in complexity when attempting to describe chemical compounds using chemical fingerprints. The relative performance of these fingerprints is discussed frequently amongst the cheminformatics community [189,190]. As much of the similarity and also fingerprint calculations did not amount to quantitative work, but rather relative comparisons, this was deemed not pertinent to the outcomes in this current study.

## 4. Conclusions

This work has provided valuable insights into the use of network strategies to investigate the distribution and drug potential of natural products within the order Verongiida. Regardless of the true origin of BTA compounds, the review and subsequent networking data investigation conducted herein have made it clear that many diverse NPs isolated from this order require further investigation.

By using bipartite network analysis together with SNs, it has been demonstrated that a variety of approaches can be utilised to display the chemical space of a set of NPs for the purpose of genera group comparison. These methods have shown that species of sponges within this order, overall and not unexpectedly, appear to follow a trend of having more similar NP chemistry with their intra-genera counterparts as opposed to their inter-genera counterparts. The comparison of secondary metabolites across the order Verongiida using networking methodology supports the current systematics of the Verongiid sponges at the family and the genus level. 

The construction of similarity networks provided the basis for discussing the chemical space and bioactivity assessment of the BTA derivatives produced by Verongiida sponges. This was also investigated using PCA analysis of pharmacokinetic chemical descriptors followed by Euclidean distance measurements. Each genus of sponge was assessed as a set of secondary metabolites as well as via cluster analysis and the drug score, showing differences across each genus in the predicted drug score. Differences in drug score were discussed via apparent chemotype and assessed using cluster analysis of the similarity networks. This showed that the genus *Verongula* is the most prolific at producing the most chemotypes with the highest mean drug score. In addition, it was shown that the BTA derivatives that contained an oxime moiety (Cluster 2) and the simple BT derivatives (Cluster 3) had the highest mean drug scores.

A noteworthy outcome of this study has been the realisation that targeted isolation strategies can be inferred from a consideration of the mean drug scores derived from an ensemble of compounds of interest. Another significant outcome of this work is the realisation that in the search for new pharmaceuticals among NP libraries, data mining, using the network analyses described herein, can provide a rational approach to the identification of likely lead candidates. While molecular networking schemes have seen more frequent use in both the cheminformatics field and the NP drug discovery field, it is apparent that the full utility of the tools provided by graph theory have yet to be fully realised.

## Figures and Tables

**Table 1:** Distribution of SIA and BT compound classes amongst Verongiida sponges (see Supporting Information S1 for references).

Species	mSIA	bSIA	mSIABT	bSIA BT	BT	BTOx	mSIAGdn	bSIAGdn	BTGdn	BTOxGdn	mSIABTOxGdn
*Aiolochroia crassa* (Hyatt, 1875)	+		+	+	+		+				
*Anomoianthella popeae* (Bergquist, 1980)					+						
*Aplysina aerophoba* (Nardo, 1833)		+		+			+				
*Aplysina archeri* (Higgin, 1875)		+		+				+			
*Aplysina caissara* (Pinheiro and Hajdu, 2001)		+					+				
*Alplysina cauliformis* (Carter, 1882)	+	+	+				+				
*Aplysina cavernicola* (Vacelet, 1959)		+		+			+				
*Aplysina fistularis* (Pallas, 1766)		+		+	+	+	+				
*Aplysina fulva* (Pallas, 1766)	+	+	+	+			+				
*Aplysina gerardogreeni* (Gomez and Bakus, 1992)		+									
*Aplysina insularis* (Duchassaing and Michelotti, 1864)	+	+		+			+				
*Aplysina lactuca* (Pinheiro, Hajdu and Custodio, 2007)		+		+							
*Aplysina lacunosa* (Lamarck, 1814)	+	+	+	+			+		+	+	+
*Aplysina laevis* (=*Pseudoceratina durissima* Carter, 1885)											
*Aplysina solongeae* (Pinheiro, Hajdu and Custodio, 2007)		+		+							
*Aplysina* sp. (Nardo, 1834)	+	+	+	+	+	+					
*Aplysina thiona* (=*Aiolochroia thiona* Laubenfels, 1930)		+									
*Aplysinella rhax* (de Laubenfels, 1954)											
*Aplysinella* sp. (Bergquist, 1980)		+	+		+					+	
*Aplysinella strongylata* (Bergquist, 1980)											
*Hexadella dedritifera* (Topsent, 1913)						+					
*Hexadella indica* (Dendy, 1905)					+						
*Hexadella* sp. (Topsent, 1896)			+		+						
*Ianthella basta* (Pallas, 1766)					+						
*Ianthella flabelliformis* (Linnaeus, 1759)			+								
*Ianthella quadrangulata* (Bergquist and Kelly-Borges, 1995)					+						
*Ianthella reticulata* (Bergquist and Kelly-Borges, 1995)											
*Ianthella* sp. (Gray, 1869)			+	+	+						
*Pseudoceratina arabica* (Keller, 1889)					+						
*Pseudoceratina crassa* (=*Aiolochroia crassa* Hyatt, 1875)	+	+	+	+	+					+	
*Pseudoceratina durissima* (Carter, 1885)		+		+							
*Pseudoceratina purea* (=*P. purpurea* Carter, 1880)	+		+		+	+	+			+	+
*Pseudoceratina purpurea* (Carter, 1880)	+		+	+	+	+	+			+	+
*Pseudoceratina* sp. (Carter, 1885)	+	+	+	+	+	+	+	+		+	+
*Pseudoceratina verrucosa* (Bergquist, 1995)	+		+	+	+	+	+		+	+	+
*Suberea clavata* (Pulitzer-Finali, 1982)							+		+	+	
*Suberea creba* (Bergquist, 1995)	+	+		+							
*Suberea ianthelliformis* (Lendenfeld, 1888)			+		+						
*Suberea mollis* (Row, 1911)		+			+		+		+		
*Suberea praetensa* (Row, 1911)				+							
*Suberea* sp. (Bergquist, 1995)	+				+	+					
*Verongula gigantea* (Hyatt, 1875)	+	+		+			+			+	
*Verongula rigida* (Esper, 1794)	+	+	+	+			+		+		
*Verongula* sp. (Verrill, 1907)	+	+		+							

**Table 2:** Murcko scaffold analysis for NPs of all genera within the order Verongiida.

Genus	Natural Products (M)	Murcko Scaffolds (N)	Singleton Scaffolds (N_sing_)	Diversity (N/M)	Novelty (N_sing_/M)
*Aiolochroia*	15	8	0	0.533	0
*Anomoianthella*	1	1	0	1	0
*Aplysina*	140	44	20	0.314	0.143
*Aplysinella*	63	19	8	0.301	0.127
*Hexadella*	12	9	0	0.75	0
*Ianthella*	95	29	20	0.305	0.211
*Pseudoceratina*	232	67	35	0.289	0.151
*Suberea*	115	47	24	0.409	0.209
*Verongula*	51	28	13	0.549	0.255

**Table 3:** PCA descriptive statistics.

	PC1	PC2	PC3	PC4	PC5
Eigenvalues	3.8916	1.2130	0.5068	0.2505	0.0841
Proportion of variance	0.649	0.202	0.084	0.014	0.009
Cumulative proportion	0.649	0.851	0.935	0.991	1.000

**Table 4:** PCA loadings for all compound descriptors.

	PC1	PC2	PC3	PC4	PC5
MW	0.472	0.147	−0.133	−0.476	−0.636
TPSA	0.458	−0.338	0.020	0.049	0.538
nHBAcc	0.471	−0.199	0.122	−0.453	0.277
nHBDon	0.420	−0.374	−0.153	0.681	−0.374
nRotB	0.307	0.522	0.748	0.270	−0.002
cLogP	0.273	0.642	−0.619	0.172	0.299

**Table 5:** Molecular descriptors for compounds that achieve a drug score ≥ 0.75.

Compound	Cluster	Drug Score	Genus	MW	TPSA	nHBAcc	nHBDon	nRotB	cLogP
**1**	3	0.81	*Aplysina,* *Suberea*	323.03	23.47	2	1	3	2.82
**2**	3	0.84	*Aplysina*	337.05	12.47	2	0	4	3.09
**3**	3	0.79	*Pseudoceratina*	380.12	38.49	3	1	7	2.63
**4**	3	0.81	*Pseudoceratina*	396.12	58.72	4	2	7	1.56
**5**	3	0.79	*Pseudoceratina*	452.19	61.8	4	2	9	2.42
**6**	3	0.78	*Pseudoceratina*	407.1	49.77	4	1	7	1.89
**7**	3	0.75	*Aplysina,* *Suberea,* *Pseudoceratina*	408.18	15.71	3	0	8	3.25
**8**	2	0.77	*Aplysina,* *Pseudoceratina*	460.13	99.6	6	4	7	2.56
**9**	2	0.88	*Aplysinella, Pseudoceratina, Verongula*	381.23	99.6	5	3	7	1.84
**10**	2	0.81	*Aplysinella*	323.23	118.2	5	3	6	0.14
**11**	2	0.8	*Pseudoceratina*	437.13	82.95	5	3	8	2.39
**12**	2	0.77	*Pseudoceratina*	475.14	118.83	7	5	7	1.84
**13**	2	0.77	*Aplysinella*	379.23	127.43	5	3	7	1.19
**14**	2	0.77	*Pseudoceratina*	460.13	99.6	5	3	7	2.56
**15**	2	0.77	*Pseudoceratina*	434.33	96.94	5	3	10	3.16
**16**	2	0.75	*Hexadella*	475.14	125.62	6	4	7	2.25
**17**	2	0.77	*Pseudoceratina*	461.11	136.62	6	5	6	1.98
**18**	2	0.89	*Pseudoceratina*	367.2	110.6	5	4	6	1.56
**19**	2	0.75	*Pseudoceratina*	475.14	125.62	7	5	7	2.25
**20**	16	0.96	*Ianthella*	254.29	63.19	3	2	1	1.01
**21**	16	0.91	*Verongula*	333.19	63.19	3	2	1	1.74
**22**	7	0.93	*Suberea*	201.61	66.4	3	2	0	−0.57
**23**	7	0.93	*Aplysina*	201.61	66.4	3	2	0	−0.57
**24**	21	0.79	*Pseudoceratina*	454.12	75.08	5	2	3	1.94
**25**	21	0.77	*Pseudoceratina*	468.15	66.29	5	1	3	2.19
**26**	42	0.94	*Pseudoceratina*	126.11	61.69	3	2	0	0.06
**27**	50	0.95	*Suberea*	154.17	97.92	4	5	2	−1.28
**28**	39	0.98	*Pseudoceratina, Verongula*	137.14	46.92	2	1	0	0.38
**29**	19	0.81	*Verongula*	346.07	19.03	1	1	3	3.2
**30**	43	0.79	*Pseudoceratina*	185.23	53.6	3	2	2	0.02
**31**	66	0.97	*Ianthella*	256.31	63.19	3	2	2	0.8
**32**	57	0.97	*Suberea*	244.29	49.41	2	1	2	0.85

MW = molecular weight, TPSA = total polar surface Area, nHBAcc = number of hydrogen bond acceptors, nHBDon = number of hydrogen bond donors, nRotB = number of rotatable bonds, cLogP = octanol/water partition coefficient.

**Table 6:** Taxonomy of sponges in the order Verongiida.

Order	Family	Genera (Total NPs)
Verongiida	Aplysinellidae	*Aplysinella* (63) *Patriciaplysina* (0) *Suberea* (115)
	Aplysinidae	*Aiolochroia* (15) *Aplysina* (140) *Verongula* (51)
	Ernstillidae	*Ernstilla* (0)
	Ianthellidae	*Anomoianthella* (1) *Hexadella* (12) *Ianthella* (95) *Vansoestia* (0)
	Pseudoceratinidae	*Pseudoceratina* (232)

## Data Availability

The data presented in this study is available in the manuscript and in the Supporting Information File.

## Supplementary Materials

The following are available online at https://www.mdpi.com/article/10.3390/md19100582/s1: Supporting Information S1: Genus Reference List, Supporting Information S2: Network Considerations, Supporting Information S3: Molecular Fingerprints, Similarity and Scaffolding, Supporting Information S4: Network Optimisation and Visualisation, Supporting Information S5: Bipartite Network Compounds vs. Species (Full Size), Supporting Information S6: Physicochemical Properties of Compounds and PCA Analysis, Supporting Information S7: References.
