# Supplementary material for: Application of Networking Approaches to Assess the Chemical Diversity, Biogeography, and Pharmaceutical Potential of Verongiida Natural Products
Source: Mar Drugs. 2021 Oct 18;19(10):582. doi: 10.3390/md19100582 (PMC8539549; doi:10.3390/md19100582)
Supplement: Supplementary file 1 [file marinedrugs-19-00582-s001.zip › New folder/marinedrugs-1412304-SI - revise.pdf]

marine drugs

Application of Networking Approaches to Assess the Chemical Diversity, Biogeography, and Pharmaceutical Potential of Verongiida Natural Products

James Lever [1], Robert Brkljača [2], Colin Rix [1] and Sylvia Urban [1,*]

[1] School of Science (Applied Chemistry and Environmental Sciences), RMIT University, GPO Box 2476V Melbourne, VIC 3001, Australia; james.lever@rmit.edu.au (J.L.); colin.rix@rmit.edu.au (C.R.)
[2] Monash Biomedical Imaging, Monash University, Clayton, VIC 3168, Australia; robert.brkljaca@monash.edu (R.B.)
* Correspondence: sylvia.urban@rmit.edu.au

Supporting Information:
S1. Genus Reference List
S2. Network Considerations
S3. Molecular Fingerprints, Similarity and Scaffolding
S4. Network Optimisation and Visualisation
S5. Bipartite Network Compounds vs Species (Full Size)
S6. Physicochemical Properties of Compounds and PCA Analysis
S7. References

S1. Genus Reference List

Table S1: Reference Table arranged by Genus.

Genus	Total NPs	References
Aiolochroia Wiedenmayer, 1977	15	[1-4]
Anomoianthella Bergquist, 1980	1	[5]
Aplysina Nardo, 1834	140	[1, 6-65]
Aplysinella Bergquist, 1980	63	[66-77]
Hexadella Topsent, 1896	12	[78-82]
Ianthella Gray, 1869	95	[83-116]
Pseudoceratina Carter, 1885	232	[25, 117-184]
Suberea Bergquist, 1995	115	[174, 185-208]
Verongula Verrill, 1907	51	[1, 6, 22, 38, 209-215]

S2. Network Considerations

The term "degree" is usually used in reference to a single node or can be considered as a mean value for the entire network. It is a count of the number of edges that are attached to a particular node. Degree usually has a strong positive correlation with the network density function (Equation 1) which can be defined as the number of edges observed in a network divided by the total number of possible edges in a network (V(V-1)/2).

$$\rho(G) = \frac{2|E|}{|V|(|V|-1)} \quad \text{(Equation 1)}$$

In most cases it is observed that when the average node degree decreases in a network, so does the network density value, as there are ultimately less edges present. This is the case when threshold values (T_c) are applied to CSNs. As T_t is increased there is always a downward trend of $\rho(G)$ [216].

Assortativity is a measure of whether a graph contains nodes that primarily connect only to other nodes with a similar degree. Graphs can have a high density and high average degree, but this is no indication as to whether nodes with high degree are grouping together or if high degree nodes only connect to low degree nodes. A network is said to be assortative when, on average, high degree nodes are connected to other high degree nodes. This concept is intrinsically linked to the homophily principle (the concept that nodes will cluster based on having similar properties to each

other) as it is an attempt at measuring the distribution of nodes within a network that are clustered due to characteristics beyond the similarity score. The value of the Degree Assortativity (Assortativity coefficient) is defined by ' r ' in Equation 2 and lies within the range -1 ≤ r ≤ 1 [217].

$$r = \frac{\sum_{ij}(e_{ij} - k_i k_j/2m)k_i k_j}{\sum_{ij}(k_i \delta_{ij} - k_i k_j/2m)k_i k_j} \quad \text{(Equation 2)}$$

Where k_i and k_j are the node degree values for the nodes i and j respectively, and m is the total number of edges in the network in question. The figure e_{ij} represents edge relationships or weighting between the nodes i and j. The value δ_{ij} is known as the Kronecker delta and is a function of the variables i and j as in Equation 3 [217].

$$\delta_{ij} = \begin{cases} 0 & if\ i \neq j \\ 1 & if\ i = j \end{cases} \quad \text{(Equation 3)}$$

Another useful metric that is often used in network analysis and optimisation is the network modularity (Q), defined by Equation 4. Modularity is a description of how strongly a given network is divided into groups of highly connected nodes that are themselves not strongly connected to other modules within the network. This idea is linked to assortativity and homophily as it is often used as the tool to measure groupings within networks, particularly in the process of clustering.

$$Q = \frac{1}{2m}\sum_{ij}\left(e_{ij}\frac{k_i k_j}{2m}\right)\delta(c_i, c_j) \quad \text{(Equation 4)}$$

Modularity optimised clustering is assigned in this study using the default Gephi cluster method, otherwise referred to as the Louvain algorithm [218]. It is measured as a function of the classes that each node is placed into using this method. These classes are represented by c_i and c_j in Equation 4, where each class is defined as an integer ranging from 1 to n_c and are held to account by the Kronecker delta of Equation 3 [217].
The average clustering coefficient (AC_c) is a global network metric that is related to the idea of modularity and graph density. The AC_c is a probability measure regarding the number of triangular groups that are created within a network. Thus, if a node N1 is connected to two nodes N2 and N3, the AC_c value indicates the probability that there will also be a connection between the nodes N2 and N3 creating a triangular group, and thus contributing towards a more densely interconnected network. Clustering coefficients are first considered on a local scale where the local clustering coefficient of a single node (C_c) is defined by Equation 5 [217].

$$C_c = \frac{2|N_i|}{k_i(k_i - 1)} \quad \text{(Equation 5)}$$

As in Equation 2, k_i refers to the degree of the node i, but the value N_i is also used, which is the number of edges that exist between the direct neighbours of the node i, thereby forming a triangular structure with the node i and two directly adjoining neighbours. This same concept can be applied to consider the average clustering coefficient of the network as a whole, where AC_c is the mean of the clustering coefficients of each node in the network.

S3. Molecular Fingerprints, Similarity and Scaffolding

Organic molecules, such as the natural products addressed in this current study, are often displayed as topographical networks of atoms, where the 3D nature of the compounds is implied. From a computational perspective however, compounds can be modelled or represented as either 2D or 3D entities. In the latter, there is a much larger amount of data quantified for theoretical molecules than in 2D illustrations. Clearly, as compound libraries get larger, the method chosen to represent compound features begins to play a larger role in the efficiency of interrogating these libraries. For this reason, all compound representations in this work are 2D global representations due to the relative simplicity and lower computational requirements of 2D models. The encoding of all the molecular structures considered in this work has been performed using SMILES codes, which is a commonly used system that allows for the representation of organic molecules in line notation and is highly compatible with many cheminformatic software packages and is often used as the basis for molecular representation [219, 220].

Cheminformatics studies involving molecular similarity as a concept are usually heavily reliant on the methodology used to describe molecular features. To assess similarity of organic compounds it is necessary to accurately describe the features of each compound and represent this information as a structure key in the form of a binary bit string or bit vector. The resultant structure key is often referred to as the molecule's fingerprint. Fingerprints are then

compared using the Tanimoto coefficient. The current work utilised the Morgan fingerprints (r=2, 2048), similar to the frequently used ECFP4 fingerprint.

S4. Network Optimisation and Visualisation

As CSN networks are created using a similarity metric (Tanimoto), the creation process requires a similarity threshold (T_t) to be applied to the network to observe useful clustering. There is, unfortunately, no ideal universal threshold value that can be applied to achieve an optimal network. This value will always be situational and specific to each data set. As a highly diverse data set may result in a higher frequency of lower Tanimoto scores requiring a very different threshold to achieve informative data compared to a data set with high frequency of larger Tanimioto score. Hence, applying the same threshold to a highly diverse data set and a nominally diverse data set will yield two networks with very different topology. The literature describes methods that have been explored using the network density property to help guide in selecting a threshold value that will appropriately represent data without compromising modularity or average clustering coefficient [216, 221]. These methods have been developed using both diverse and non-diverse compound data sets that have been applied to small organic compounds. These studies observed that a good approximation of the appropriate network density value, leading to desirable network characteristics can be made between 2.5-5.0 % [216, 221]. The methodology adopted for threshold selection involved optimisation of key network statistics (network degree assortativity, clustering coefficient, transitivity and network density, **Figure 1**, to achieve an appropriate threshold and density value **Figure 2**. Observation of key network statistics showed that the threshold value of 0.5 results in a local maximum of network transitivity as well as degree assortativity two statistics that display the promotion of homophily within the networks as well as significant local clustering at this threshold. This threshold also results in a minimum number of isolates (6.9% of nodes) as well as maintaining a network density close to the literature suggested value of 5.0% (6.2%).

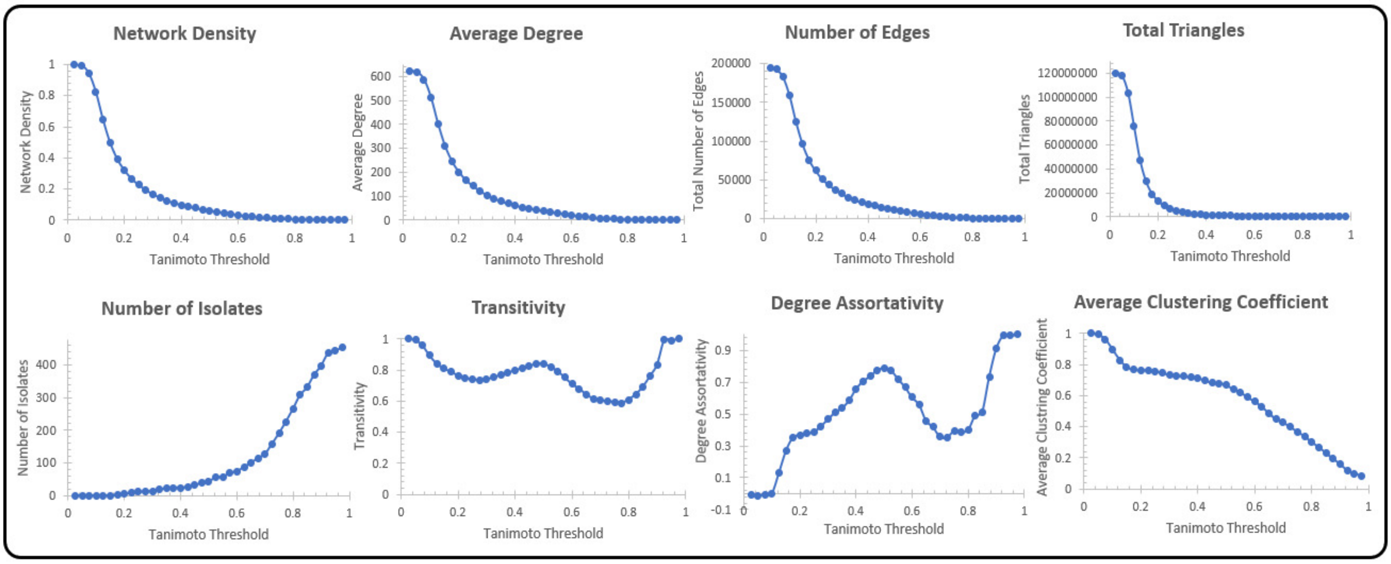

Figure S1: Network statistics for CSN (Morgan2, 2048).

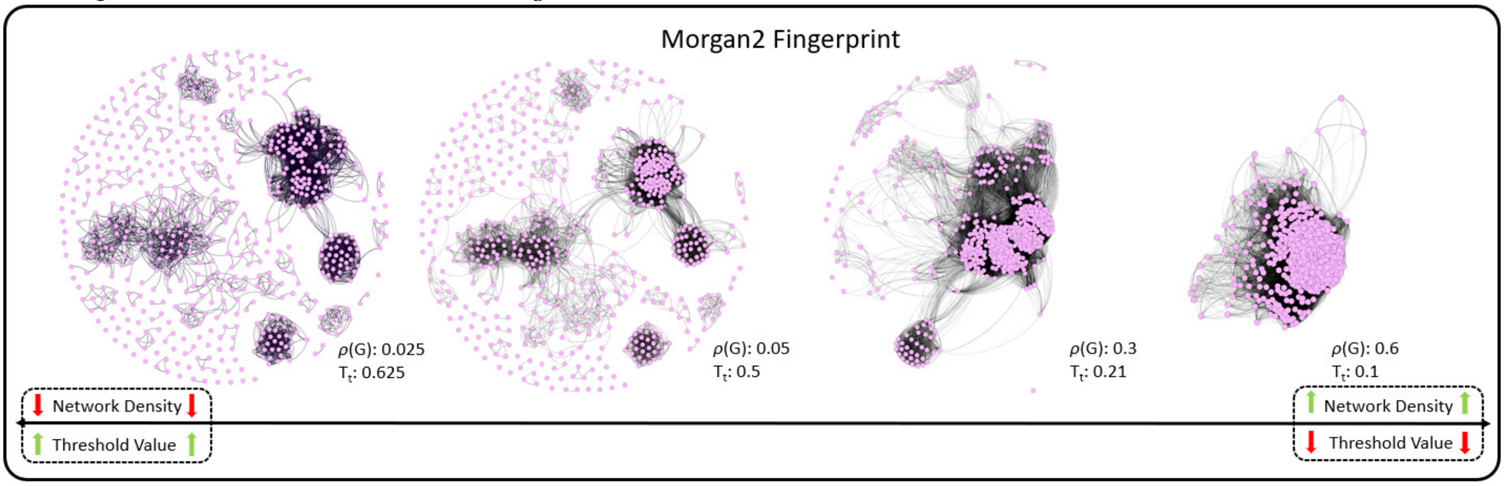

Figure S2: Networks displayed at differing Tanimoto threshold values (T_t) using Morgan2 fingerprints.

S5. Bipartite Network Compounds vs Species (Full Size).

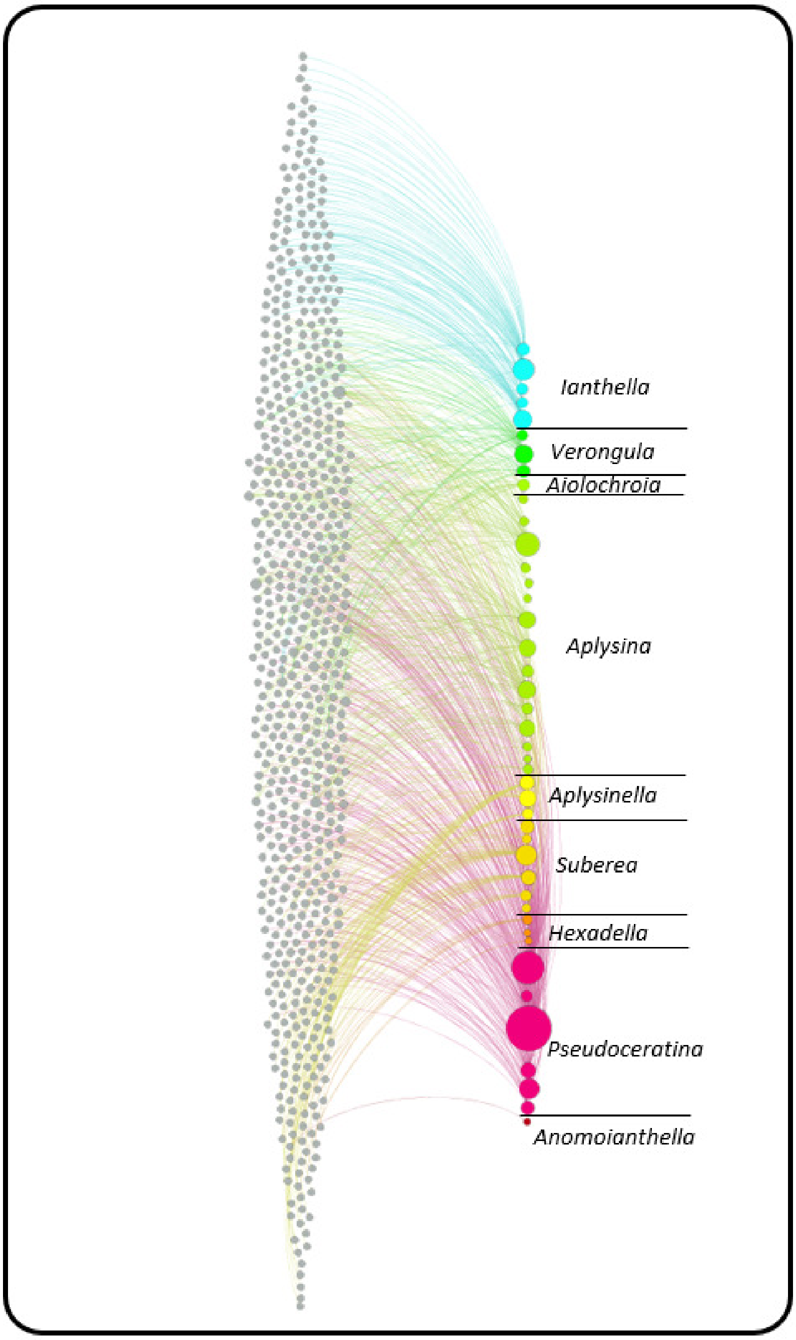

Figure S3: Simple Bipartite Network used to create monopartite projection of compound sharing with species arranged via genus.

S6. Physicochemical Properties of Compounds and PCA Analysis

All physicochemical properties (**Figures 4-7**) for compounds were calculated using the OSIRIS property explorer followed by PCA analysis using Minitab version 19.2.

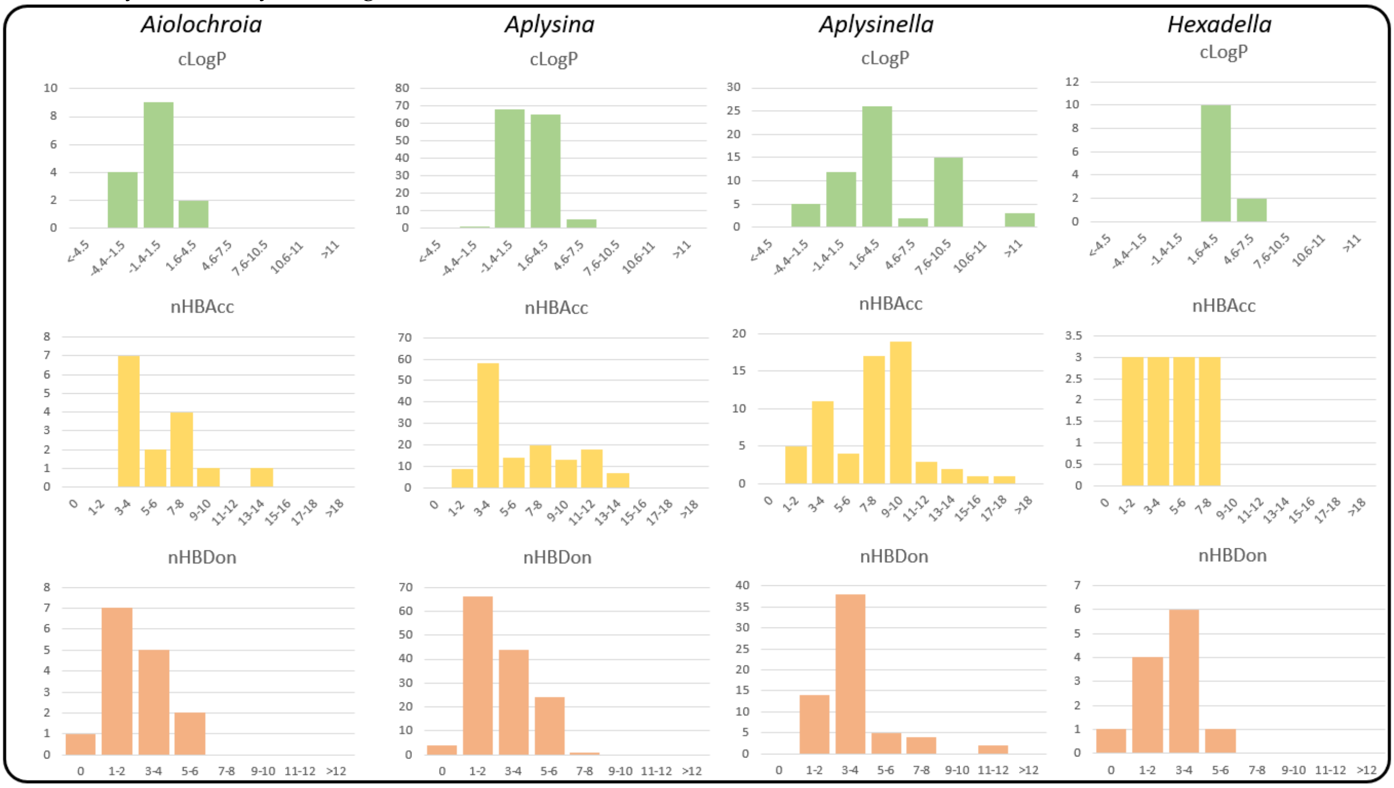

Figure S4: Physicochemical properties (cLogP, nHBAcc and nHBDon) for NPs from *Aiolochroia*, *Aplysina*, *Aplysinella* and *Hexadella*.

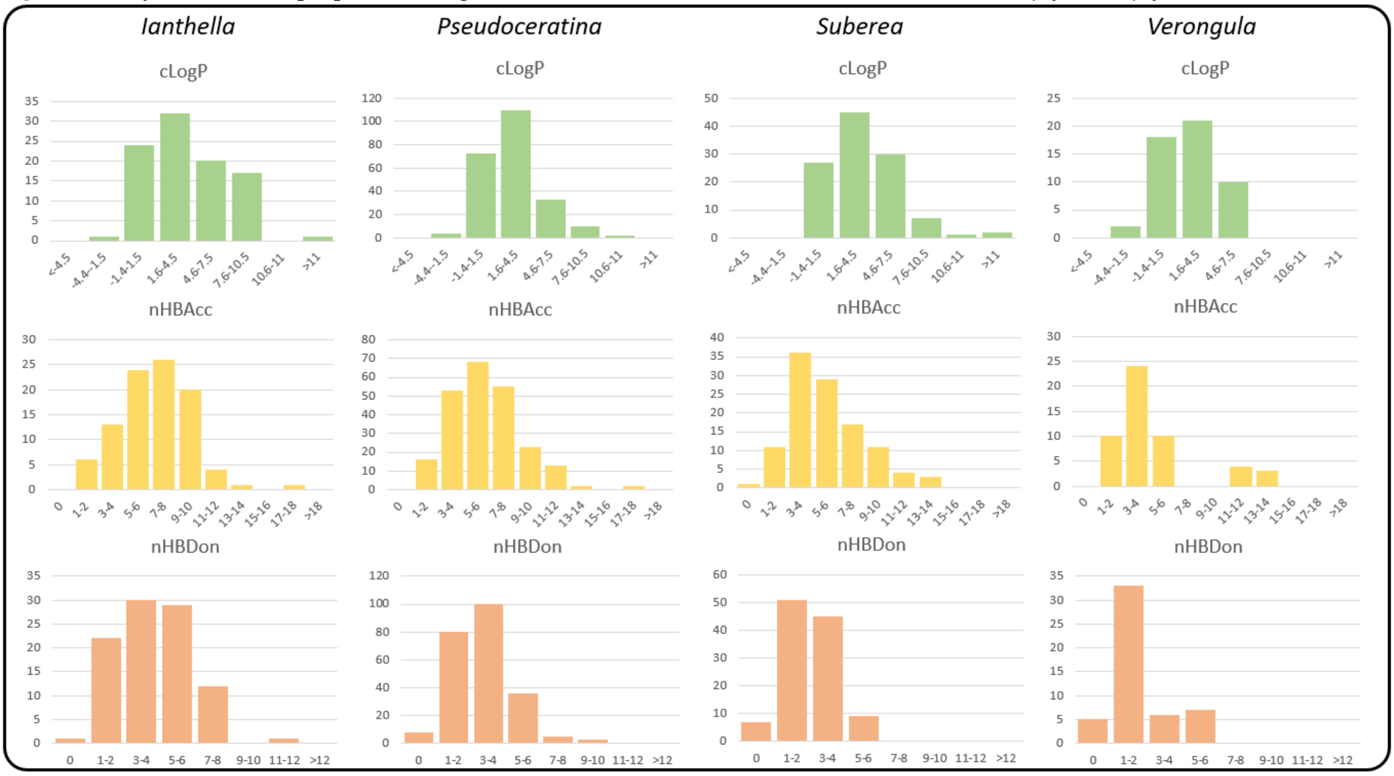

Figure S5: Physicochemical properties (cLogP, nHBAcc and nHBDon) for NPs from *Ianthella*, *Pseudoceratina*, *Suberea* and *Verongula*.

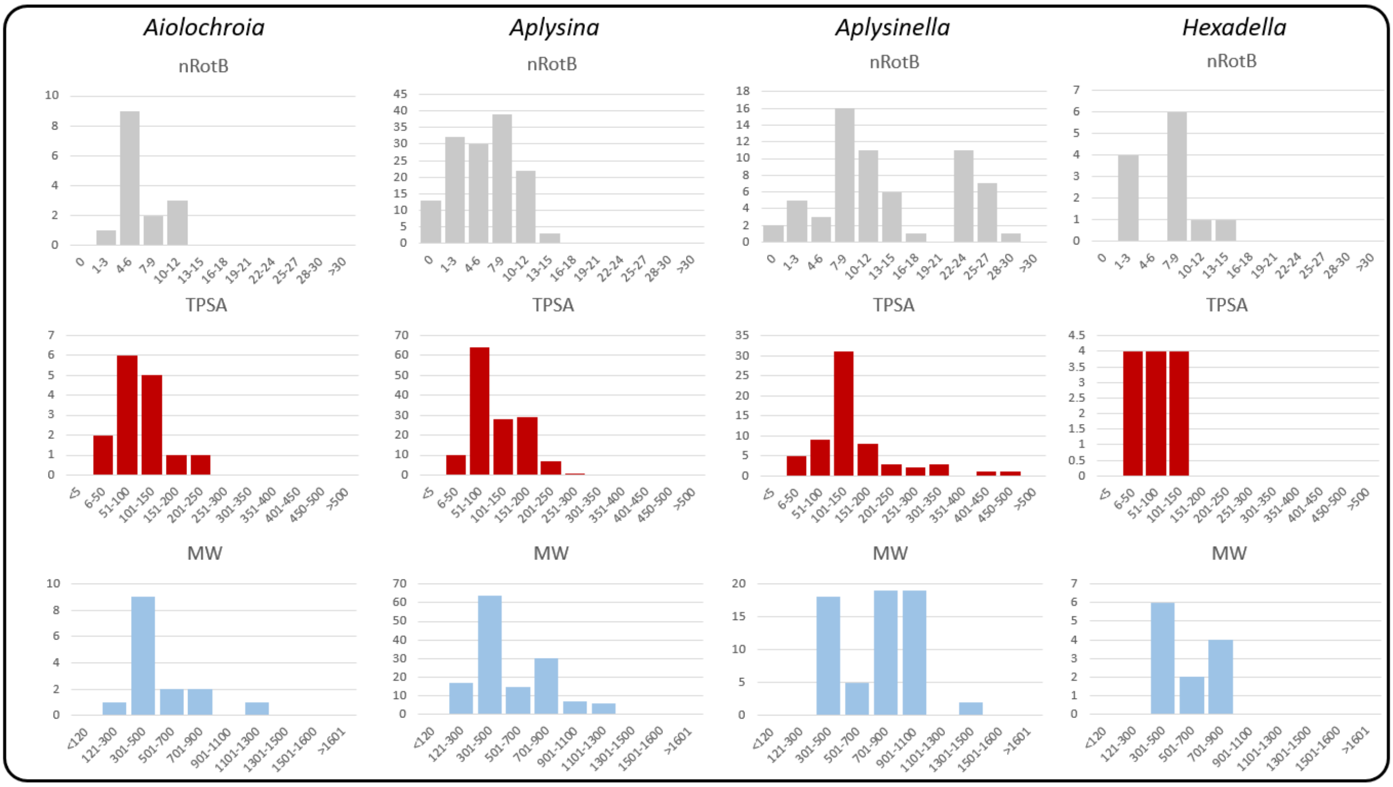

Figure S6: Physicochemical properties (nRotB, TPSA and MW) for NPs from *Aiolochroia*, *Aplysina*, *Aplysinella* and *Hexadella*.

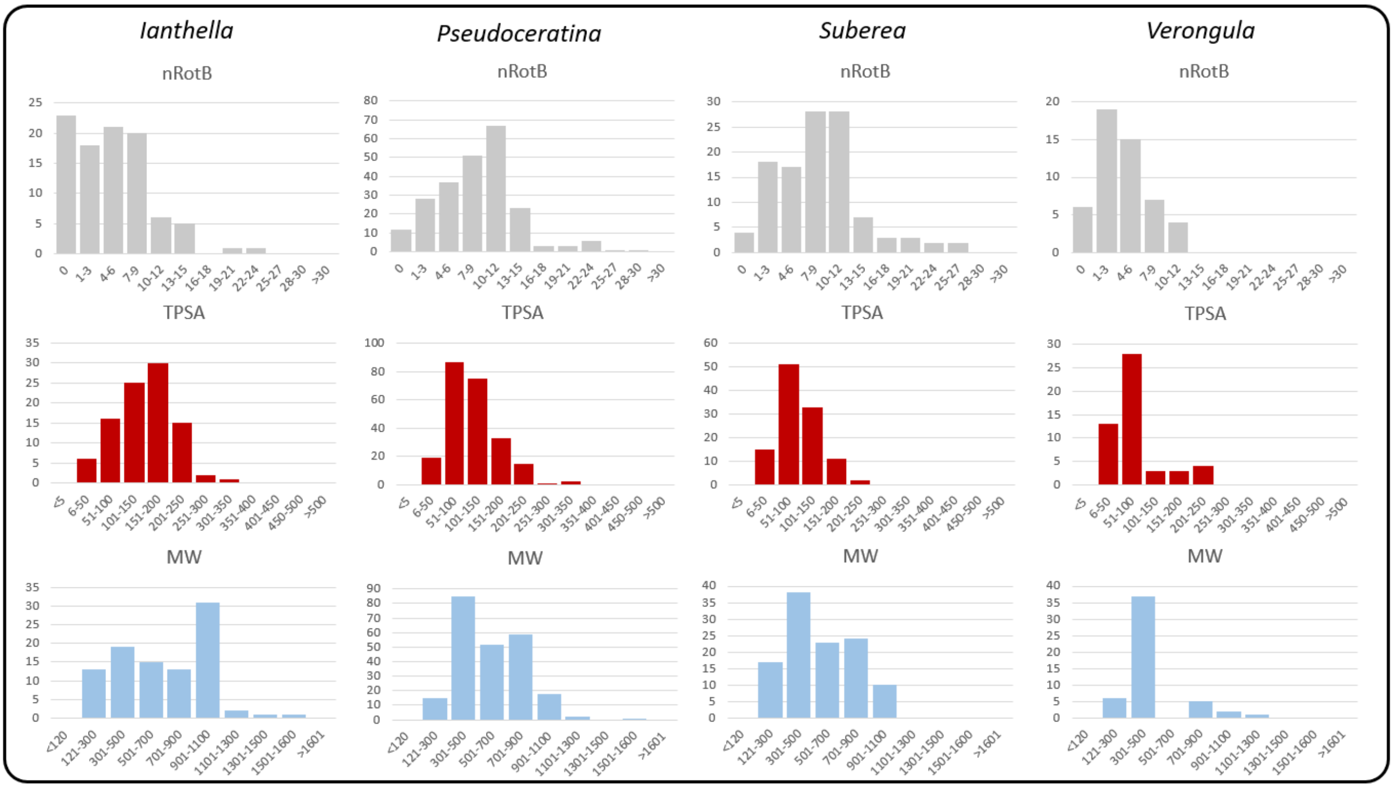

Figure S7: Physicochemical properties (nRotB, TPSA and MW) for NPs from *Ianthella*, *Pseudoceratina*, *Suberea* and *Verongula*

S7. References

1. Makarieva, T.N.; Stonik, V.A.; Alcolado, P. and Elyakov, Y.B., Comparative study of the halogenated tyrosine derivatives from Demospongiae (Porifera). *Comp. Biochem. Physiol.*, **1981**. *68B*, p. 481-484.
2. Assmann, M.; Wray, V.; van Soest, R.W.M. and Proksch, P., A new bromotyrosine alkaloid from the Caribbean sponge *Aiolochroia crassa. Z. Naturforsch.*, **1998**. *53c*, p. 398-401.
3. Gao, H.; Kelly, M. and Hamann, M.T., Bromotyrosine-derived metabolites from the sponge *Aiolochroia crassa. Tetrahedron*, **1999**. *55*, p. 9717-9726.
4. Galeano, E.; Martinez, A.; Thomas, O.P.; Robledo, S. and Munoz, D., Antiparasitic bromotyrosine derivatives from the Caribbean marine sponge *Aiolochroia crassa. Quim. Nova.*, **2012**. *35*(6), p. 1189-1193.
5. Kernan, M.R. and Cambie, R.C., Chemistry of Sponges, VIII. 1 Anomoian A, A bromotyrosine derivative from *Anomoianthella popeae. J. Nat. Prod.*, **1990**. *53*(3), p. 720-723.
6. Fattorusso, E.; Minale, L. and Sodano, G., Aeroplysinin-I, a New Bromo-compound from *Aplysina aerophoba. Chem. Comm.*, **1970**. *12*, p. 751-752.
7. Sharma, G.M.; Vig, B. and Burkholder, P.R., Antimicrobial substances of sponges. IV. Structure of a bromine-containing compound from a marine sponge. *J. Org. Chem.*, **1970**. *35*(8), p. 2823-2826.
8. Fattorusso, E.; Minale, L. and Sodano, G., Aeroplysinin-1, an antibacterial bromo-compound from the sponge *Verongia aerophoba. J. Chem. Soc.*, **1972**, p. 16-18.
9. Borders, D.B.; Morton, G.O. and Wetzel, E.R., Structure of a novel bromine compound isolated from a sponge. *Tetrahedron Lett.*, **1974**. *31*, p. 2709-2712.
10. Krejcarek, G.E.; White, R.H.; Hager, L.P.; McClure, W.O.; Johnson, R.D.; Rinehart, K.L.; McMillan, J.A. and Paul, I.C., A rearranged dibromotyrosine metabolite from *Verongia aurea. Tetrahedron Lett.*, **1975**. *8*, p. 507-510.
11. Gopichand, Y. and Schmitz, F.J., Marine natural products fistularin-1, -2 and -3 from the sponge *Aplysina fistularis* forma *fulva. Tetrahedron Lett.*, **1979**. *41*, p. 3921-3924.
12. D'Ambrosio, M.; Guerriero, A.; Traldi, P. and Pietra, F., Cavernicolin-1 and cavernicolin-2, two epimeric dibromolactams from the Mediterranean sponge *Aplysina* (*Verongia*) *cavernicola. Tetrahedron Lett.*, **1982**. *23*(42), p. 4403-4406.
13. Cimino, G.; De Rosa, S.; De Stefano, S.; Self, R. and Sodano, G., The bromo-compounds of the true sponge *Verongia aerophoba. Tetrahedron Lett.*, **1983**. *24*(29), p. 3029-3032.
14. D'Ambrosio, M.; Guerriero, A.; De Clauser, R.; De Stanchina, G. and Pietra, F., Dichloroverongiaquinol, a new marine antibacterial compound from *Aplysina cavernicola*. Isolation and synthesis. *Experentia*, **1983**. *39*, p. 1091-1092.
15. D'Ambrosio, M.; Guerriero, A. and Pietra, F., Novel, racemic or nearly-racemic antibacterial bromo- and chloroquinols and Lactams of the verongiaquinol and the cavernicolin type from the marine sponge *Aplysina* (=*Verongia*) *cavernicola. Helv. Chim. Acta*, **1984**. *67*, p. 1484-1492.
16. Goo, Y.M., Antimicrobial and antineoplastic tyrosine metabolites from a marine sponge, *Aplysina fistularis. Arch. Pharm. Res.*, **1985**. *8*(1), p. 21-30.
17. Walker, R.P.; Thompson, J.E. and Faulkner, D.J., Exudation of biologically-active metabolites in the sponge *Aplysina fistularis. Mar. Biol.*, **1985**. *88*, p. 27-32.
18. D'Ambrosio, M.; Mealli, C.; Guerriero, A. and Pietra, F., 7-Bromocavernicolenone, a New Bromoenone from the Mediterranean sponge *Aplysina* (=*Verongia*) *cavernicola*. Implied, unprecendented involvement of a halogenated dopa in the biogenesis of a natural product. *Helv. Chim. Acta*, **1985**. *68*, p. 1453-1460.
19. Capon, R.J. and MacLeod, J.K., Two Epimeric Dibromo Nitriles From the Australian Sponge *Aplysina laevis. Aust. J. Chem.*, **1987**. *40*, p. 341-346.
20. Rodriguez, A.D.; Akee, R.K. and Scheur, P.J., Two bromotyrosine-cysteine derived metabolites from a sponge. *Tetrahedron Lett.*, **1987**. *28*(42), p. 4989-4992.

21. Morte, M.; Rodriguez, M.L.; Fernandez, J.J.; Eguren, L. and Estrade, D.M., Aplysinadiene and (r,r) 5 [3,5-dibromo-4-[(2-oxo-5-oxazolidinyl)] methoxyphenyl]-2-oxazolidinone, two novel metabolites from *Aplysina aerophoba* synthes. *Tetrahedron*, **1988**. *44*(15), p. 4973-4980.
22. Gunasekera, M. and Gunasekera, S.P., Dihydroxyaerothionin and aerophobin 1. Two brominated tyrosine metabolites from the deep water marine sponge *Verongula rigida. J. Nat. Prod.*, **1989**. *52*(4), p. 753-756.
23. Xynas, R. and Capon, R.J., Two New Bromotyrosine-Derived Metabolites From an Australian Marine Sponge, *Aplysina* sp. *Aust. J. Chem.*, **1989**. *42*, p. 1427-1433.
24. Cruz, F.; Quijano, L.; Gomez-Garibay, F. and Rios, T., Brominated Metabolites from the Sponge *Aplysina* (*verongia*) *thiona. J. Nat. Prod.*, **1990**. *53*(3), p. 543-548.
25. Kobayashi, J.; Tsuda, M.; Agemi, K.; Shigemori, H.; Ishibashi, M.; Sasaki, T. and Mikami, Y., Purealidins B and C new bromotyrosine alkaloids from the okinawan marine sponge *Psammplysilla purea. Tetrahedron*, **1991**. *47*(33), p. 6617-6622.
26. Gunaserka, S.P. and Cross, S.S., Fistularin 3 and 11-ketofistularin 3. Feline leukemia virus active bromotyrosine metabolites from the marine sponge *Aplysina archeri. J. Nat. Prod.*, **1992**. *55*(4), p. 509-512.
27. Acosta, A.I. and Rodriguez, A.D., 11-Oxoaerothionin A Cytotoxic Antitumor Bromotyrosine-Derived Alkaloid from the Caribbean Marine Sponge *Aplysina lacunosa. J. Nat. Prod.*, **1992**. *55*(7), p. 1007-1012.
28. Rodriguez, A.D. and Pina, I.C., The structures of aplysinamisines I, II, and III New bromotyrosine-derived alkaloids from the Caribbean sponge *Aplysina cauliformi. J. Nat. Prod.*, **1993**. *56*(6), p. 907-914.
29. Mancini, I.; Guella, G.; Laboute, P.; Debitus, C. and Pietra, F., Hemifistularin 3: a Degraded Peptide or Biogenetic Precursor? Isolation from a sponge of the order Verongida from the Coral Sea or generation from base treatment of 11-Oxofistularin 3. *J. Chem. Soc. Perkin Trans.*, **1993**. *24*, p. 3121-3125.
30. Ciminiello, P.; Costantino, V.; Fattorusso, E.; Magno, S. and Mangoni, A., Chemistry of Verongida sponges, II. Constituents of the Caribbean sponge *Aplysina fistularis forma fulva. J. Nat. Prod.*, **1994**. *57*(6), p. 705-712.
31. Carney, J.R. and Rinehart, K.L., Biosynthesis of Brominated Tyrosine Metabolites by *Aplysina fistularis. J. Nat. Prod.*, **1995**. *58*(7), p. 971-985.
32. Ciminiello, P.; Dell'Aversano, C.; Fattorusso, E. and Magno, S., Chemistry of Verongida sponges. VII Bromocompounds from the caribbean sponge *Aplysina archeri. Tetrahedron*, **1996**. *52*(29), p. 9863-9868.
33. Ciminiello, P.; Fattorusso, E.; Forino, M. and Magno, S., Chemistry of Verongida sponges VIII Bromocompounds from the Mediterranean sponges *Aplysina aerophoba* and *Aplysina cavernicola. Tetrahedron*, **1997**. *18*, p. 6565-6572.
34. Ciminiello, P.; Dell'Aversano, C.; Fattorusso, E.; Magno, S. and Pansini, M., Chemistry of Verongida Sponges. 9. Secondary Metabolite Composition of the Caribbean Sponge *Aplysina cauliformis. J. Nat. Prod.*, **1999**. *62*, p. 590-593.
35. Fendert, T.; Wray, V.; van Soest, R.W.M. and Proksch, P., Bromoisoxazoline Alkaloids from the Caribbean Sponge *Aplysina insularis. Z. Naturforsch.*, **1999**. *54c*, p. 246-252.
36. Compagnone, R.S.; Avila, R.; Suarez, A.I.; Abrams, O.V.; Rangel, H.R.; Arvelo, F.; Pina, I.C. and Merentes, E., 11-Deoxyfistularin-3, a New Cytotoxic Metabolite from the Caribbean Sponge *Aplysina fistularis insularis. J. Nat. Prod.*, **1999**. *62*, p. 1443-1444.
37. Encarnacion, R.D.; Sandoval, E.; Malmstrom, J. and Christophersen, C., Calafianin, a Bromotyrosine Derivative from the Marine Sponge *Aplysina gerardogreeni. J. Nat. Prod.*, **2000**. *63*, p. 874-875.
38. Ciminiello, P.; Dell'Aversano, C.; Fattorusso, E.; Magno, S. and Pansini, M., Chemistry of Verongida Sponges. 10. Secondary Metabolite Composition of the Caribbean Sponge *Verongula gigantea. J. Nat. Prod.*, **2000**. *63*, p. 263-266.
39. Ciminiello, P.; Dell'Aversano, C.; Fattorusso, E. and Magno, S., Archerine, a Novel Anti-Histaminic Bromotyrosine-Derived Compound from the Caribbean Marine Sponge *Aplysina archeri. Eur. J. Org. Chem.*, **2001**, p. 55-60.
40. Evan, T.; Rudi, A.; Ilan, M. and Kashman, Y., Aplyzanzine A, a New Dibromotyrosine Derivative from a Verongida Sponge. *J. Nat. Prod.*, **2001**. *64*, p. 226-227.
41. Saeki, B.M.; Granata, A.C.; Berlinck, R.G.S.; Magalhaes, A.; Schefer, A.B.; Ferreira, A.G.; Pinheiro, U.S. and Hajdu, E., Two Unprecedented Dibromotyrosine-Derived Alkaloids from the Brazilian Endemic Marine Sponge *Aplysina caissara. J. Nat. Prod.*, **2002**. *65*, p. 796-799.

42. Rogers, E.W.; Fernanda de Oliveira, M.; Berlinck, R.G.S.; Konig, G.M. and Molinski, T.F., Stereochemical Heterogeneity in Verongid Sponge Metabolites. Absolute Stereochemistry of (+)-Fistularin-3 and (+)-11-epi-Fistularin-3 by Microscale LCMS-Marfey's Analysis. *J. Nat. Prod.*, **2005**. *68*, p. 891-896.
43. de Lira, T.O.; Berlinck, R.G.S.; Nascimento, G.G.F. and Hajdu, E., Further dibromotyrosine-derived metabolites from the marine sponge *Aplysina caissara. J. Braz. Chem. Soc.*, **2006**. *17*(7), p. 1233-1240.
44. Rogers, E.W. and Molinski, T.F., Highly polar spiroisoxazolines from the sponge *Aplysina fulva. J. Nat. Prod.*, **2007**. *70*, p. 1191-1194.
45. Hernandez-Guerrero, C.J.; Zubia, E.; Ortega, M.J. and Carballo, J.L., Cytotoxic dibromotyrosine-derived metabolites from the sponge *Aplysina gerardogreeni. Bioorg. Med. Chem.*, **2007**. *15*(15), p. 5275-5282.
46. Kossuga, M.H.; de Lira, S.P.; Nascimento, A.M.; Gambardella, M.T.P.; Berlinck, R.G.S.; Torres, Y.R.; Nascimento, G.G.F.; Pimenta, E.F.; Silva, M.; Thiemann, O.H., et al., Isolamento e atividades biologicas de produtos naturais das esponjas monanchora arbuscula, *Aplysina* sp. *Quim. Nova.*, **2007**. *30*, p. 1194-1202.
47. Nuñez, C.V.; de Almeida, E.V.R.; Granato, A.C.; Marques, S.O.; Santos, K.O.; Pereira, F.R.; Macedo, M.L.; Ferreira, A.G.; Hajdu, E.; Pinheiro, U.S., et al., Chemical variability within the marine sponge *Aplysina fulva. Biochem. Syst. and Ecol.*, **2008**. *36*(4), p. 283-296.
48. Santalova, E.A.; Denisenko, V.A. and Stonik, V.A., Dibromotyrosine and histamine derivatives from the tropical marine sponge *Aplysina* sp. *Nat. Prod. Comm.*, **2010**. *5*(3), p. 377-382.
49. Silva, M.M.; Bergamasco, J.; Lira, S.P.; Lopes, N.P.; Hajdu, E.; Peixinho, S. and Berlinck, R.G.S., Dereplication of bromotyrosine-derived metabolites by LC-PDA-MS and analysis of the chemical profile of 14 *Aplysina* sponge specimens from the Brazilian coastline. *Aust. J. Chem.*, **2010**. *63*, p. 886-894.
50. Gandolfi, R.C.; Medina, M.B.; Berlinck, R.G.S.; Lira, S.P.; de Sa Galetti, F.C.; Silva, C.L.; Veloso, K.; Ferreira, A.G.; Hajdu, E. and Peixinho, S., metabolitos secundarios das esponjas *Aplysina fistularis* e *dysidea* sp. e atividade antituberculose da 11-cetofistularina-3. *Quim. Nova.*, **2010**. *33*(9), p. 1852-1858.
51. Sacristan-Soriano, O.; Banaigs, B. and Becerro, M.A., Relevant spatial scales of chemical variation in *Aplysina aerophoba. Mar. Drugs*, **2011**. *9*(12), p. 2499-2513.
52. Santalova, E.A.; Denisenko, V.A.; Glazunov, V.P.; Kalinovskii, A.I.; Anastyuk, S.D. and Stonik, V.A., Dibromotyrosine derivatives from the ethanol extract of the marine sponge *Aplysina* sp. structures transformations and origin. *Russ. Chem. Bull.*, **2011**. *60*(3), p. 570-580.
53. de Morais Lima, G.R.; de Albuquerque Montenegro, C.; de Almeida, C.L.; de Athayde-Filho, P.F.; Barbosa-Filho, J.M. and Batista, L.M., Database survey of anti-inflammatory plants in South America: a review. *Int. J. Mol. Sci.*, **2011**. *12*(4), p. 2692-2749.
54. Sacristan-Soriano, O.; Banaigs, B. and Becerro, M.A., Temporal trends in the secondary metabolite production of the sponge *Aplysina aerophoba. Mar. Drugs*, **2012**. *10*(4), p. 677-693.
55. Lira, N.S.; Monte-Neto, R.L.; Marchi, J.G.B.; da Silva Lins, A.C.; Tavares, J.F.; da Silva, M.S.; da Silva Dias, C.; Barbosa-Filho, J.M.; dos Santos, C.F.; da Cunha, E.V.L., et al., Aplysfistularine: a novel dibromotyrosine derivative isolated from *Aplysina fistularis. Quim. Nova.*, **2012**. *35*(11), p. 2189-2193.
56. de Medeiros, A.I.; Gandolfi, R.C.; Secatto, A.; Falcucci, R.M.; Faccioli, L.H.; Hajdu, E.; Peixinho, S. and Berlinck, R.G., 11-Oxoaerothionin isolated from the marine sponge *Aplysina fistularis* shows anti-inflammatory activity in LPS-stimulated macrophages. *Immunopharmacol. Immunotoxicol.*, **2012**. *34*(6), p. 919-924.
57. Santalova, E.A.; Denisenko, V.A. and Dmitrenok, P.S., Minor brominated compounds from extract of the sponge *Aplysina* sp. *Chem. Nat. Comp.*, **2013**. *49*(1), p. 75-78.
58. Kunze, K.; Niemann, H.; Ueberlein, S.; Schulze, R.; Ehrlich, H.; Brunner, E.; Proksch, P. and van Pee, K.H., Brominated skeletal components of the marine demosponges, *Aplysina cavernicola* and *Ianthella basta*: analytical and biochemical investigations. *Mar. Drugs*, **2013**. *11*(4), p. 1271-1287.
59. Gallimore, W.A., Bioactive brominated metabolites from the natural habitat and tank maintained cuttings of the Jamaican sponge *Aplysina fistularins. Chem. Biodivers.*, **2013**. *10*, p. 1055-1060.

60. Puyana, M.; Pawlik, J.; Blum, J. and Fenical, W., Metabolite variability in Caribbean sponges of the genus *Aplysina. Rev. Bras. Farmacogn.*, **2015**. *25*(6), p. 592-599.
61. Gothel, Q.; Sirirak, T. and Kock, M., Bromotyrosine-derived alkaloids from the Caribbean sponge *Aplysina lacunosa. Beilstein. J. Org. Chem.*, **2015**. *11*, p. 2334-2342.
62. Reverter, M.; Perez, T.; Ereskovsky, A.V. and Banaigs, B., Secondary Metabolome Variability and Inducible Chemical Defenses in the Mediterranean Sponge *Aplysina cavernicola. J. Chem. Ecol.*, **2016**. *42*(1), p. 60-70.
63. Binnewerg, B.; Schubert, M.; Voronkina, A.; Muzychka, L.; Wysokowski, M.; Petrenko, I.; Djurovic, M.; Kovalchuk, V.; Tsurkan, M.; Martinovic, R., et al., Marine biomaterials: Biomimetic and pharmacological potential of cultivated *Aplysina aerophoba* marine Demosponge. *Mater. Sci. Eng. C. Mater. Biol. Appl.*, **2020**. *109*, p. 110566.
64. Salib, M.N.; Jamison, M.T. and Molinski, T.F., Bromo-spiroisoxazoline Alkaloids, Including an Isoserine Peptide, from the Caribbean Marine Sponge *Aplysina lacunosa. J. Nat. Prod.*, **2020**. *83*(5), p. 1532-1540.
65. Lira, N.S.; Montes, R.C.; Tavares, J.F.; da Silva, M.S.; da Cunha, E.V.; de Athayde-Filho, P.F.; Rodrigues, L.C.; da Silva Dias, C. and Barbosa-Filho, J.M., Brominated compounds from marine sponges of the genus *Aplysina* and a compilation of their ^{13}C NMR spectral data. *Mar. Drugs*, **2011**. *9*(11), p. 2316-2368.
66. Ichiba, T. and Scheuer, P.J., Three Bromotyrosine Derivatives, One Terminating in an Unprecedented Diketocyclopentenylidene Enamine. *J. Org. Chem*, **1993**. *58*, p. 4149-4150.
67. Fu, X. and Schmitz, F.J., 7-Hydroxyceratinamine, a New Cyanoformamide-Containing Metabolite from a Sponge, *Aplysinella sp. J. Nat. Prod.*, **1999**. *62*, p. 1072-1073.
68. Pham, N.B.; Butler, M.S. and Quinn, R.J., Isolation of Psammaplin A 11′-Sulfate and Bisaprasin 11′-Sulfate from the Marine Sponge *Aplysinella rhax. J. Nat. Prod.*, **2000**. *63*, p. 393-395.
69. Shin, J.; Lee, H.-S.; Seo, Y.; Rho, J.-R.; Cho, K.W. and Paul, V.J., New Bromotyrosine Metabolites from the Sponge *Aplysinella rhax. Tetrahedron*, **2000**. *56*, p. 9071-9077.
70. Tabudravu, J.N.; Eijsink, V.G.H.; Gooday, G.W.; Jaspars, M.; Komander, D.; Legg, M.; Synstad, B. and van Aalten, D.M.F., Psammaplin A, a chitinase inhibitor isolated from the Fijian marine sponge *Aplysinella rhax. Bioorg. Med. Chem.*, **2002**. *10*, p. 1123-1128.
71. Thoms, C. and Schupp, P.J., Activated chemical defense in marine sponges--a case study on *Aplysinella rhax. J. Chem. Ecol.*, **2008**. *34*(9), p. 1242-1252.
72. Ankudey, F.J.; Kiprof, P.; Stromquist, E.R. and Chang, L.C., New bioactive bromotyrosine-derived alkaloid from a marine sponge *Aplysinella* sp. *Planta Med.*, **2008**. *74*(5), p. 555-559.
73. Graham, S.K.; Lambert, L.K.; Pierens, G.K.; Hooper, J.N.A. and Garson, M.J., Psammaplin Metabolites New and Old: An NMR Study Involving Chiral Sulfur Chemistry. *Aust. J. Chem.*, **2010**. *63*, p. 867-872.
74. Mudianta, I.W.; Skinner-Adams, T.; Andrews, K.T.; Davis, R.A.; Hadi, T.A.; Hayes, P.Y. and Garson, M.J., Psammaplysin derivatives from the Balinese marine sponge *Aplysinella strongylata. J. Nat. Prod.*, **2012**. *75*(12), p. 2132-2143.
75. Tian, L.W.; Feng, Y.; Shimizu, Y.; Pfeifer, T.; Wellington, C.; Hooper, J.N. and Quinn, R.J., Aplysinellamides A-C, bromotyrosine-derived metabolites from an Australian *Aplysinella* sp. marine sponge. *J. Nat. Prod.*, **2014**. *77*(5), p. 1210-1214.
76. Mudianta, I.W., Bioprospecting of the Balinese marine sponges and nudibranchs. *J. Phys. Conf. Ser.*, **2018**. *1040*, p. 1-7.
77. Shaala, L.A. and Youssef, D.T.A., Cytotoxic Psammaplysin Analogues from the Verongid Red Sea Sponge *Aplysinella* Species. *Biomolecules*, **2019**. *9*(12), p. 1-10.
78. Morris, S.A. and Anderson, R.J., Nitrogenous metabolites from the deep water sponge *Hexadella* sp. *Can. J. Chem.*, **1989**. *67*, p. 677-681.
79. Morris, S.A. and Anderson, R.J., Brominated bis(indole) alkaloids from the marine sponge *Hexadella* sp. *Tetrahedron*, **1990**. *46*(3), p. 715-720.
80. Matsunaga, S.; Kobayashi, H.; van Soest, R.W.M. and Fusetani, N., Novel bromotyrosine derivatives that inhibit growth of the fish pathogenic bacterium *Aeromonas hydrophila*, from a marine sponge *Hexadella* sp. *J. Org. Chem.*, **2005**. *70*, p. 1893-1896.

81. Cardenas, P., Who Produces Ianthelline? The Arctic Sponge Stryphnus fortis or its Sponge Epibiont *Hexadella dedritifera*: a Probable Case of Sponge-Sponge Contamination. *J. Chem. Ecol.*, **2016**. *42*(4), p. 339-347.
82. Tarazona, G.; Santamaria, G.; Cruz, P.G.; Fernandez, R.; Perez, M.; Martinez-Leal, J.F.; Rodriguez, J.; Jimenez, C. and Cuevas, C., Cytotoxic Anomoian B and Aplyzanzine B, New Bromotyrosine Alkaloids from Indonesian Sponges. *ACS Omega*, **2017**. *2*(7), p. 3494-3501.
83. Minale, L.; Sodano, G.; Chan, W.R. and Chen, A.M., Aeroplysinin-2, a Dibromolactone from marine sponges *Aplysina* (Verongia) *aerophoba* and *Ianthella* sp. *J. C. S. Chem. Comm.*, **1972**, p. 674-675.
84. Kazlauskas, R.; Lidgard, R.O.; Murphy, P.T.; Wells, R.J. and Blount, J.F., Brominated tyrosine-derived metabolites from the sponge *Ianthella basta. Aust. J. Chem.*, **1981**. *34*, p. 765-786.
85. Miao, S. and Anderson, R.J., Cytotoxic metabolites from the sponge *Ianthella basta* collected in Papua New Guinea. *J. Nat. Prod.*, **1990**. *53*(6), p. 1441-1446.
86. Pordesimo, E.O. and Schmitz, F.J., New bastadins from the sponge *Ianthella basta. J. Org. Chem.*, **1990**. *55*(15), p. 4704-4709.
87. Butler, M.S.; Lim, T.K.; Capon, R.J. and Hammond, L.S., The Bastadins Revisited: New Chemistry From the Australian Marine Sponge *Ianthella basta. Aust. J. Chem.*, **1991**. *44*, p. 287-296.
88. Gulavita, N.K.; Wright, A.E.; McCarthy, P.J.; Pomponi, S.A. and Kelly-Borges, M., Isolation and structure elucidation of 34-sulfatobastadin 13, an inhibitor of the endothelin a receptor, from a marine sponge of the genus *Ianthella. J. Nat. Prod.*, **1993**. *56*(9), p. 1613-1617.
89. Dexter, A.F. and Garson, M.J., Isolation of a novel bastadin from the temperate marine sponge *Ianthella* sp. *J. Nat. Prod.*, **1993**. *56*(5), p. 782-786.
90. Mack, M.M.; Molinski, T.F.; Buck, E.D. and Pessah, I.N., Novel modulators of skeletal muscle FKBP12/calcium channel complex from *Ianthella basta. J. Biol. Chem.*, **1994**. *269*(37), p. 23236-23249.
91. Jaspars, M.; Rali, T.; Laney, M.; Schatzman, R.C.; Diaz, M.C.; Schmitz, F.J.; Pordesimo, E.O. and Crews, P., The search for inosine 5′-Phosphate dehydrogenase (IMPDH) inhibitors from marine sponges. Evaluation of the bastadin alkaloids. *Tetrahedron*, **1994**. *50*(25), p. 7367-7374.
92. Park, S.K.; Jurek, J.; Carney, J.R. and Scheuer, P.J., Two more bastadins, 16 and 17, from an Indonesian sponge *Ianthella basta. J. Nat. Prod.*, **1994**. *57*(3), p. 407-410.
93. Pettit, G.R.; Butler, M.S.; Bass, C.G.; Doubek, D.L.; Williams, M.D.; Schmidt, J.M.; Pettit, R.K.; Hooper, J.N.A.; Tackett, L.P. and Filiatrault, M.J., Antineoplastic agents, 326. The stereochemistry of bastadins 8, 10, and 12 from the Bismarck Archipelago marine sponge *Ianthella basta. J. Nat. Prod.*, **1995**. *58*(5), p. 680-688.
94. Park, S.K.; Park, H. and Scheuer, P.J., Isolation and structure determination of a new bastadin from an Indonesian sponge *Ianthella basta. Bull. Korean Chem. Soc.*, **1994**. *15*(7), p. 534-537.
95. Franklin, M.A.; Penn, S.G.; Lebrilla, C.B.; Lam, T.H.; Pessah, I.N. and Molinski, T.F., Bastadin 20 and Bastadin O-Sulfate Esters from *Ianthella basta*: Novel Modulators of the Ry1R FKBP12 Receptor Complex. *J. Nat. Prod.*, **1996**. *59*, p. 1121-1127.
96. Pettit, G.R.; Butler, M.S.; Williams, M.D.; Filiatrault, M.J. and Pettit, R.K., Isolation and Structure of Hemibastadinols 1−3 from the Papua New Guinea Marine Sponge *Ianthella basta. J. Nat. Prod.*, **1996**. *59*, p. 927-934.
97. Okamoto, Y.; Ojika, M. and Sakagami, Y., Iantheran A, a dimeric polybrominated benzofuran as a Na,K-ATPase inhibitor from a marine sponge, *Ianthella sp. Tetrahedron Lett.*, **1999**. *40*, p. 507-510.
98. Okamoto, Y.; Ojika, M.; Kato, S. and Sakagami, Y., Ianthesines A–D, Four Novel Dibromotyrosine-Derived Metabolites from a Marine Sponge, *Ianthella sp. Tetrahedron*, **2000**. *56*, p. 5813-5818.
99. Okamoto, Y.; Ojika, M.; Suzuki, S.; Murakami, M. and Sakagami, Y., Iantherans A and B, unique dimeric polybrominated benzofurans as Na, K-ATPase inhibitors from a marine sponge, *Ianthella sp. Bioorg. Med. Chem.*, **2001**. *9*, p. 179-183.
100. Coll, J.C.; Kearns, P.S.; Rideout, J.A. and Sankar, V., Bastadin 21, a Novel Isobastarane Metabolite from the Great Barrier Reef Marine Sponge *Ianthella quadrangulata. J. Nat. Prod.*, **2002**. *65*, p. 753-756.

101. Masuno, M.N.; Hoepker, A.C.; Pessah, I.N. and Molinski, T.F., 1-*O*-Sulfatobastadins-1 and -2 from *Ianthella basta* (Pallas). Antagonists of the RyR1-FKBP12 Ca^{2+} Channel. *Mar. Drugs*, **2004**. *2*, p. 176-184.
102. Aoki, S.; Cho, S.H.; Hiramatsu, A.; Kotoku, N. and Kobayashi, M., Bastadins, cyclic tetramers of brominated-tyrosine derivatives, selectively inhibit the proliferation of endothelial cells. *J. Nat. Med.*, **2006**. *60*(3), p. 231-235.
103. Greve, H.; Meis, S.; Kassack, M.U.; Kehraus, S.; Krick, A.; Wright, A.D. and Konig, G.M., New Iantherans from the Marine Sponge *Ianthella quadrangulata* Novel Agonists of the P2Y11 Receptor. *J. Med. Chem.*, **2007**. *50*, p. 5600-5607.
104. Greve, H.; Kehraus, S.; Krick, A.; Kelter, G.; Maier, A.; Fiebig, H.-H.; Wright, A.D. and Konig, G.M., Cytotoxic Bastadin 24 from the Australian Sponge *Ianthella quadrangulata. J. Nat. Prod.*, **2008**. *71*, p. 309-312.
105. Motti, C.A.; Freckelton, M.L.; Tapiolas, D.M. and Willis, R.H., FTICR-MS and LC-UV/MS-SPE-NMR Applications for the rapid dereplication of a crude extract from the sponge *Ianthella flabelliformis. J. Nat. Prod.*, **2009**. *72*, p. 290-294.
106. Carroll, A.R.; Kaiser, S.M.; Davis, R.A.; Moni, R.W.; Hooper, J.N.A. and Quinn, R.J., A Bastadin with Potent and Selective δ-Opioid Receptor Binding Affinity from the Australian Sponge *Ianthella flabelliformis. J. Nat. Prod.*, **2010**. *73*, p. 1173-1176.
107. Calcul, L.; Inman, W.D.; Morris, A.A.; Tenney, K.; Ratnam, J.; McKerrow, J.H.; Valeriote, F.A. and Crews, P., Additional insights on the bastadins: Isolation of analogues from the sponge *Ianthella* cf. *reticulata* and exploration of the oxime configurations. *J. Nat. Prod.*, **2010**. *73*, p. 365-372.
108. Zhang, H.; Conte, M.M.; Huang, X.C.; Khalil, Z. and Capon, R.J., A search for BACE inhibitors reveals new biosynthetically related pyrrolidones, furanones and pyrroles from a southern Australian marine sponge, *Ianthella* sp. *Org. Biomol. Chem.*, **2012**. *10*(13), p. 2656-2663.
109. Feng, Y.; Bowden, B.F. and Kapoor, V., Ianthellamide A, a selective kynurenine-3-hydroxylase inhibitor from the Australian marine sponge *Ianthella quadrangulata. Bioorg. Med. Chem. Lett.*, **2012**. *22*(10), p. 3398-3401.
110. Zhang, H.; Conte, M.M.; Khalil, Z.; Huang, X.-C. and Capon, R.J., New dictyodendrins as BACE inhibitors from a southern Australian marine sponge, *Ianthella* sp. *RSC Advances*, **2012**. *2*(10), p. 4209-4214.
111. Balansa, W.; Islam, R.; Gilbert, D.F.; Fontaine, F.; Xiao, X.; Zhang, H.; Piggott, A.M.; Lynch, J.W. and Capon, R.J., Australian marine sponge alkaloids as a new class of glycine-gated chloride channel receptor modulator. *Bioorg. Med. Chem.*, **2013**. *21*(14), p. 4420-4425.
112. Niemann, H.; Lin, W.; Muller, W.E.; Kubbutat, M.; Lai, D. and Proksch, P., Trimeric hemibastadin congener from the marine sponge *Ianthella basta. J. Nat. Prod.*, **2013**. *76*(1), p. 121-125.
113. Mathieu, V.; Wauthoz, N.; Lefranc, F.; Niemann, H.; Amighi, K.; Kiss, R. and Proksch, P., Cyclic versus hemi-bastadins. pleiotropic anticancer effects: from apoptosis to anti-angiogenic and anti-migratory effects. *Molecules*, **2013**. *18*(3), p. 3543-3561.
114. Huang, X.C.; Xiao, X.; Zhang, Y.K.; Talele, T.T.; Salim, A.A.; Chen, Z.S. and Capon, R.J., Lamellarin O, a pyrrole alkaloid from an Australian marine sponge, *Ianthella* sp., reverses BCRP mediated drug resistance in cancer cells. *Mar. Drugs*, **2014**. *12*(7), p. 3818-3837.
115. Eguchi, K.; Kato, H.; Fujiwara, Y.; Losung, F.; Mangindaan, R.E.; de Voogd, N.J.; Takeya, M. and Tsukamoto, S., Bastadins, brominated-tyrosine derivatives, suppress accumulation of cholesterol ester in macrophages. *Bioorg. Med. Chem. Lett.*, **2015**. *25*(22), p. 5389-5392.
116. Gartshore, C.J.; Salib, M.N.; Renshaw, A.A. and Molinski, T.F., Isolation of bastadin-6-O-sulfate and expedient purifications of bastadins-4, -5 and -6 from extracts of *Ianthella basta. Fitoterapia*, **2018**. *126*, p. 16-21.
117. Nakamura, H.; Wu, H.; Kobayashi, J.; Nakamura, Y.; Ohizumi, Y. and Hirata, Y., Purealin a novel enzyme activator from the okinawan marine sponge *Psammaplysilla purea. Tetrahedron Lett.*, **1985**. *26*(37), p. 4517-4520.
118. Roll, D.M.; Chang, C.W.J.; Scheur, P.J.; Gray, G.A.; Shoolery, J.N.; Matsumoto, G.K.; Van Duyne, G.D. and Clardy, J., Structure of the Psammaplysins. *J. Am. Chem. Soc.*, **1985**. *107*, p. 2916-2920.
119. Quinoa, E. and Crews, P., Phenolic constituents of *Psammaplysilla. Tetrahedron Lett.*, **1987**. *28*(28), p. 3229-3232.
120. Jimenez, C. and Crews, P., Novel marine sponge derived amino acids 13. additional psammaplin derivatives from *Psammplysilla purpurea. Tetrahedron*, **1991**. *47*(12/13), p. 2097-2102.
121. James, D.M.; Kunze, H.B. and Faulkner, D.J., Two new brominated tyrosine derivatives from the sponge *Druinella purpurea. J. Nat. Prod.*, **1991**. *54*(4), p. 1137-1140.

122. Copp, B.R. and Ireland, C.M., Psammaplysin C a new cytotoxic dibromotyrosine derived metabolite from the marine sponge *Druinella purpurea. J. Nat. Prod.*, **1992**. *55*(6), p. 822-823.
123. Tsuda, M.; Shigemori, H.; Ishibashi, M. and Kobayashi, J., Purealidin D a new pyridine alkaloid from the Okinawan marine sponge *Psammplysilla purea. Tetrahedron Lett.*, **1992**. *33*(18), p. 2597-2598.
124. Tsuda, M.; Shigemori, H.; Ishibashi, M. and Kobayashi, J., Purealidins E-G new bromotyrosine alkaloids from the Okinawan marine sponge *Psammaplysilla purea. J. Nat. Prod.*, **1992**. *55*(9), p. 1325-1327.
125. Carney, J.R.; Scheur, P.J. and Kelly-Borges, M., A new bastadin from the sponge *Psammaplysilla purpurea. J. Nat. Prod.*, **1993**. *56*(1), p. 153-157.
126. Yagi, H.; Matsunaga, S. and Fusetani, N., Purpuramines A-I, New bromotyrosine-derived metabolites from the marine sponge *Psammaplysilla purpurea. Tetrahedron*, **1993**. *49*(18), p. 3749-3754.
127. Jurek, J.; Yoshida, W.Y. and Scheur, P.J., Three new bromotyrosine derived metabolites of the sponge *Psammaplysilla purpurea. J. Nat. Prod.*, **1993**. *56*(9), p. 1609-1612.
128. Pakrashi, S.C.; Achari, B.; Dutta, P.K.; Chakrabarti, A.K.; Giri, C. and Saha, S., Marine products from bay of bengal constituents of the sponge *Psammplysilla purpurea. Tetrahedron*, **1994**. *50*(41), p. 12009-12014.
129. Honma, K.; Tsuda, M.; Mikami, Y. and Kobayashi, J.i., Aplysillamides A and B, new antimicrobial guanidine alkaloids from the Okinawan marine sponge *Psammaplysilla purea. Tetrahedron*, **1995**. *51*(13), p. 3745-3748.
130. Venkateswarlu, Y. and Chavakula, R., Brominated benzeneacetonitriles, the dibromotyrosine metabolites from the sponge *Psammaplysilla purpurea. J. Nat. Prod.*, **1995**. *58*(7), p. 1087-1088.
131. Kobayashi, J.; Honma, K. and Tsuda, M., Lipopurealins D and E and purealidin H new bromotyrosine alkaloids from the okinawan marine sponge *Psammplysilla purea. J. Nat. Prod.*, **1995**. *58*(3), p. 467-470.
132. Kobayashi, J.; Honma, K. and Tsuda, M., Purealidins J-R new bromotyrosine alkaloids from the Okinawan marine sponge *Pammplysilla purea. Chem. Pharm. Bull.*, **1995**. *43*(3), p. 403-407.
133. Venkateswarlu, Y.; Rama Roa, M. and Venkatesham, U., A new dibromotyrosine derived metabolite from the sponge *Psammplysilla purpurea. J. Nat. Prod.*, **1998**. *61*, p. 1388-1389.
134. Venkateswarlu, Y.; Venkatesham, U. and Rama Roa, M., Novel Bromine containing constituents of the sponge *Psammplysilla purpurea. J. Nat. Prod.*, **1999**. *62*, p. 893-894.
135. Rama Roa, M.; Venkatesham, U. and Venkateswarlu, Y., Two bromo compounds from the sponge *Psammaplysilla purpurea. Ind. J. Chem.*, **1999**. *38B*, p. 1301-1303.
136. Venkateshwar Goud, T.; Srinivasulu, M.; Reddy, V.L.N.; Reddy, A.V.; Rao, T.P.; Kumar, D.S.; Murty, U.S. and Venkateswarlu, Y., Two new bromotyrosine derived metabolites from the sponge *Psammaplysilla purpurea. Chem. Pharm. Bull.*, **2003**. *51*(8), p. 990-993.
137. Tilvi, S.; Rodrigues, C.; Naik, C.G.; Parameswaran, P.S. and Wahidhulla, S., New bromotyrosine alkaloids from the marine sponge *Psammaplysilla purpurea. Tetrahedron*, **2004**. *60*(45), p. 10207-10215.
138. Ravinder, K.; Vijender Reddy, A.; Raju, T.V. and Venkateswarlu, Y., A new dibromotyrosine derived metabolite from the sponge *Psammaplysilla purpurea. ARKIVOC*, **2005**, p. 51-55.
139. Schroeder, F.C.; Kau, T.R.; Silver, P.A. and Clardy, J., The psammaplysenes specific inhibitors of FOXO1a Nuclear export. *J. Nat. Prod.*, **2005**. *68*, p. 574-576.
140. Fujiwara, T.; Hwang, J.H.; Kanamoto, A.; Nagai, H.; Takagi, M. and Shin-Ya, K., JBIR-44, a new bromotyrosine compound from a marine sponge *Psammaplysilla purpurea. J. Antibiot. (Tokyo)*, **2009**. *62*(7), p. 393-395.
141. Tilvi, S. and D'Souza, L., Identifying the related compounds using electrospray ionization tandem mass spectrometry: bromotyrosine alkaloids from marine sponge *Psammaplysilla purpurea. Eur. J. Mass Spectrom. (Chichester)*, **2012**. *18*(3), p. 333-343.
142. Tilvi, S. and Majik, M.S., 2D NMR Studies of Bromotyrosine Alkaloid, Purpurealidin K from Marine Sponge *Psammaplysilla purpurea. ChemistrySelect*, **2019**. *4*(21), p. 6568-6571.

143. Martin James, D.; Kunze, H.B. and Faulkner, D.J., Two new brominated tyrosine derivatives from the sponge *Druinella purpurea. J. Nat. Prod.*, **1991**. *54*(4), p. 1137-1140.

144. Tabudravu, J.N. and Jaspars, M., Purealidin S and purpuramine J Bromotyrosine alkaloids from the Fijian marine sponge *Druinella* sp. *J. Nat. Prod.*, **2002**. *65*, p. 1798-1801.

145. Kassuhlke, K.E. and Faulkner, J.D., Two new dibromotyrosine derivatives from the caribbean sponge *Pseudoceratina crassa. Tetrahedron*, **1991**. *47*(10/11), p. 1809-1814.

146. Kernan, M.R. and Cambie, R.C., Chemistry of sponges, VII. 11, 19-dideoxyfistularin 3 and 11-hydroxyaerothionin, bromotyrosine derivatives from *Pseudoceratina durissima. J. Nat. Prod.*, **1990**. *53*(3), p. 615-622.

147. Albrizio, S.; Ciminiello, P.; Fattorusso, E. and Magno, S., Chemistry of Verongida sponges. I. constituents of the caribbean sponge *Pseudoceratina crassa. Tetrahedron*, **1994**. *50*(3), p. 783-788.

148. Ciminiello, P.; Fattorusso, E. and Magno, S., Chemistry of Verongida sponges, IV. comparison of the secondary metabolite composition of several specimens of *Pseudoceratina crassa. J. Nat. Prod.*, **1995**. *58*(5), p. 689-696.

149. Aiello, A.; Fattorusso, E.; Menna, M. and Pansini, M., Chemistry of Verongida sponges V. Brominated metabolites from the Caribbean sponge *Pseudoceratina* sp. *Biochem. Syst. and Ecol.*, **1995**. *23*(4), p. 377-381.

150. Benharref, A. and Pais, M., Bromotyrosine alkaloids from the sponge *Pseudoceratina verrucosa. J. Nat. Prod.*, **1996**. *59*, p. 177-180.

151. Tsukamoto, S.; Kato, H.; Hirota, H. and Fusetani, N., Ceratinamides A and B: New antifouling dibromotyrosine derivatives from the marine sponge *Pseudoceratina purpurea. Tetrahedron*, **1996**. *52*(24), p. 8181-8186.

152. Tsukamoto, S.; Kato, H.; Hirota, H. and Fusetani, N., Ceratinamine: An unprecedented antifouling cyanoformamide from the marine sponge *Pseudoceratina purpurea. J. Org. Chem.*, **1996**. *61*, p. 2936-2937.

153. Tsukamoto, S.; Kato, H.; Hirota, H. and Fusetani, N., Pseudoceratidine a new antifouling spermidine derivative from the marine sponge *Pseudoceratina purpurea. Tetrahedron Lett.*, **1996**. *37*(9), p. 1439-1440.

154. Thirionet, I.; Daloze, D.; Braekman, J.C. and Willemsen, P., 5-Bromoverongamine, a Novel Antifouling Tyrosine Alkaloid from the Sponge *Pseudoceratina* sp. *Nat. Prod. Lett.*, **1998**. *12*(3), p. 209-214.

155. Fusetani, N.; Masuda, Y.; Nakao, Y.; Matsunaga, S. and van Soest, R.W.M., Three new bromotyrosine derivatives lethal to crab from the marine sponge *Pseudoceratina purpurea. Tetrahedron*, **2001**. *57*, p. 7507-7511.

156. Takada, N.; Watanabe, R.; Suenaga, K.; Yamada, K.; Ueda, K.; Kita, M. and Uemura, D., Zamamistatin a significant antibacterial bromotyrosine derivative, from the Okinawan sponge *Pseudoceratina purpurea. Tetrahedron Lett.*, **2001**. *42*, p. 5265-5267.

157. Manzo, E.; van Soest, R.; Matainaho, L.; Roberge, M. and Anderson, R.J., Ceratamines A and B, antimitotic heterocyclic alkaloids isolated from the marine sponge *Pseudoceratina* sp collected in Papua New Guinea. *Org. Lett.*, **2003**. *5*(24), p. 4591-4594.

158. Pina, I.C.; Gautschi, J.T.; Wang, G.; Sanders, M.L.; Schmitz, F.J.; France, D.; Cornell-Kennon, S.; Sambucetti, L.C.; Remiszewski, S.W.; Perez, L.B., et al., Psammaplins from the sponge *Pseudoceratina purpurea* inhibition of both histone deacetylase and DNA methyltransferase. *J. Org. Chem.*, **2003**. *68*, p. 3866-3873.

159. Kijjoa, A.; Bessa, J.; Wattanadilok, R.; Sawangwong, P.; Nascimento, N.S.J.; Pedro, M.; Silva, A.M.S.; Eaton, G.; van Soest, R. and Herz, W., Dibromotyrosine derivatives, a maleimide, aplysamine-2 and other constituents of the marine sponge *Pseudoceratina purpurea. Z. Naturforsch.*, **2005**. *60b*, p. 904-908.

160. Hayakawa, I.; Teruya, T. and Kigoshi, H., Revised structure of zamamistatin. *Tetrahedron Lett.*, **2006**. *47*(2), p. 155-158.

161. Jang, J.H.; van Soest, R.W.M.; Fusetani, N. and Matsunaga, S., Pseudoceratins A and B, antifungal bicyclic bromotyrosine-derived metabolites from the marine sponge *Pseudoceratina purpurea. J. Org. Chem.*, **2007**. *72*, p. 1211-1217.

162. Buchanan, M.S.; Carroll, A.R.; Fechner, G.A.; Boyle, A.; Simpson, M.M.; Addepalli, R.; Avery, V.M.; Hooper, J.N.; Su, N.; Chen, H., et al., Spermatinamine, the first natural product inhibitor of isoprenylcysteine carboxyl methyltransferase, a new cancer target. *Bioorg. Med. Chem. Lett.*, **2007**. *17*(24), p. 6860-6863.

163. Buchanan, M.S.; Carroll, A.R.; Fechner, G.A.; Boyle, A.; Simpson, M.; Addepalli, R.; Avery, V.M.; Hooper, J.N.A.; Cheung, T.; Chen, H., et al., Aplysamine 6, alkaloid inhibitor of isoprenylcysteine carboxyl methyltransferase from the sponge *Pseudoceratina* sp. *J. Nat. Prod.*, **2008**. *71*, p. 1066-1067.

164. Badr, J.M.; Shaala, L.A.; Abou-Shoer, M.I.; Tawfik, M.K. and Habib, A.-A.M., Bioactive brominated metabolites from the Red Sea sponge *Pseudoceratina arabica. J. Nat. Prod.*, **2008**. *71*, p. 1472-1474.

165. Huang, X.-P.; Deng, Z.-W.; van Soest, R.W.M. and Lin, W.-H., Brominated derivatives from the Chinese sponge *Pseudoceratina* sp. *J. Asian Nat. Prod. Res.*, **2008**. *10*(3), p. 239-242.

166. Kalaitzis, J.A.; Leone Pde, A.; Hooper, J.N. and Quinn, R.J., Ianthesine E, a new bromotyrosine-derived metabolite from the Great Barrier Reef sponge *Pseudoceratina* sp. *Nat. Prod. Res.*, **2008**. *22*(14), p. 1257-1263.

167. Ma, K.; Yang, Y.; Deng, Z.; de Voogd, N.J.; Proksch, P. and Lin, W., Two new bromotyrosine derivatives from the marine sponge *Pseudoceratina* sp. *Chem. Biodivers.*, **2008**. *5*, p. 1313-1320.

168. Lebouvier, N.; Jullian, V.; Desvignes, I.; Maurel, S.; Parenty, A.; Dorin-Semblat, D.; Doerig, C.; Sauvain, M. and Laurent, D., Antiplasmodial activities of homogentisic acid derivative protein kinase inhibitors isolated from a Vanuatu marine sponge *Pseudoceratina* sp. *Mar. Drugs*, **2009**. *7*(4), p. 640-653.

169. McCulloch, M.W.; Coombs, G.S.; Banerjee, N.; Bugni, T.S.; Cannon, K.M.; Harper, M.K.; Veltri, C.A.; Virshup, D.M. and Ireland, C.M., Psammaplin A as a general activator of cell-based signaling assays via HDAC inhibition and studies on some bromotyrosine derivatives. *Bioorg. Med. Chem.*, **2009**. *17*(6), p. 2189-2198.

170. Kon, Y.; Kubota, T.; Shibazaki, A.; Gonoi, T. and Kobayashi, J., Ceratinadins A-C, new bromotyrosine alkaloids from an Okinawan marine sponge *Pseudoceratina* sp. *Bioorg. Med. Chem. Lett.*, **2010**. *20*(15), p. 4569-4572.

171. Feng, Y.; Davis, R.A.; Sykes, M.L.; Avery, V.M.; Camp, D. and Quinn, R.J., Pseudoceratinazole A: a novel bromotyrosine alkaloid from the Australian sponge *Pseudoceratina* sp. *Tetrahedron Lett.*, **2010**. *51*(37), p. 4847-4850.

172. Xu, M.; Andrews, K.T.; Birrell, G.W.; Tran, T.L.; Camp, D.; Davis, R.A. and Quinn, R.J., Psammaplysin H, a new antimalarial bromotyrosine alkaloid from a marine sponge of the genus *Pseudoceratina. Bioorg. Med. Chem. Lett.*, **2011**. *21*(2), p. 846-848.

173. Yin, S.; Davis, R.A.; Shelper, T.; Sykes, M.L.; Avery, V.M.; Elofsson, M.; Sundin, C. and Quinn, R.J., Pseudoceramines A-D, new antibacterial bromotyrosine alkaloids from the marine sponge *Pseudoceratina* sp. *Org. Biomol. Chem.*, **2011**. *9*(19), p. 6755-6760.

174. Shaala, L.A.; Youssef, D.T.; Sulaiman, M.; Behery, F.A.; Foudah, A.I. and Sayed, K.A., Subereamolline A as a potent breast cancer migration, invasion and proliferation inhibitor and bioactive dibrominated alkaloids from the Red Sea sponge *Pseudoceratina arabica. Mar. Drugs*, **2012**. *10*(11), p. 2492-2508.

175. Salim, A.A.; Khalil, Z.G. and Capon, R.J., Structural and stereochemical investigations into bromotyrosine-derived metabolites from southern Australian marine sponges, *Pseudoceratina* spp. *Tetrahedron*, **2012**. *68*(47), p. 9802-9807.

176. Tran, T.D.; Pham, N.B.; Fechner, G.; Hooper, J.N. and Quinn, R.J., Bromotyrosine alkaloids from the Australian marine sponge *Pseudoceratina verrucosa. J. Nat. Prod.*, **2013**. *76*(4), p. 516-523.

177. Su, J.H.; Chen, Y.C.; El-Shazly, M.; Du, Y.C.; Su, C.W.; Tsao, C.W.; Liu, L.L.; Chou, Y.; Chang, W.B.; Su, Y.D., et al., Towards the small and the beautiful: a small dibromotyrosine derivative from *Pseudoceratina* sp. sponge exhibits potent apoptotic effect through targeting IKK/NFkappaB signaling pathway. *Mar. Drugs*, **2013**. *11*(9), p. 3168-3185.

178. Olatunji, O.J.; Ogundajo, A.L.; Oladosu, I.A.; Changwichit, K.; Ingkaninan, K.; Yuenyongsawad, S. and Plubrukarn, A., Non-competitive inhibition of acetylcholinesterase by bromotyrosine alkaloids. *Nat. Prod. Comm.*, **2014**. *9*(11), p. 1559-1561.

179. Shaala, L.A.; Youssef, D.T.A.; Badr, J.M.; Sulaiman, M.; Khedr, A. and El Sayed, K.A., Bioactive alkaloids from the Red Sea marine Verongid sponge *Pseudoceratina arabica. Tetrahedron*, **2015**. *71*(41), p. 7837-7841.

180. Gotsbacher, M.P. and Karuso, P., New antimicrobial bromotyrosine analogues from the sponge *Pseudoceratina purpurea* and its predator *Tylodina corticalis. Mar. Drugs*, **2015**. *13*(3), p. 1389-1409.

181. Ragini, K.; Fromont, J.; Piggott, A.M. and Karuso, P., Enantiodivergence in the Biosynthesis of Bromotyrosine Alkaloids from Sponges? *J. Nat. Prod.*, **2017**. *80*(1), p. 215-219.

182. Rahelivao, M.P.; Lubken, T.; Gruner, M.; Kataeva, O.; Ralambondrahety, R.; Andriamanantoanina, H.; Checinski, M.P.; Bauer, I. and Knolker, H.J., Isolation and structure elucidation of natural products of three soft corals and a sponge from the coast of Madagascar. *Org. Biomol. Chem.*, **2017**. *15*(12), p. 2593-2608.

183. Kurimoto, S.I.; Ohno, T.; Hokari, R.; Ishiyama, A.; Iwatsuki, M.; Omura, S.; Kobayashi, J. and Kubota, T., Ceratinadins E and F, New Bromotyrosine Alkaloids from an Okinawan Marine Sponge *Pseudoceratina* sp. *Mar. Drugs*, **2018**. *16*(12), p. 1-7.

184. Li, H.; Yu, H.; Wu, W. and Sun, P., Chemical constituents of sponge *Pseudoceratina* sp. and their chemotaxonomic significance. *Biochem. Syst. and Ecol.*, **2020**. *89*.

185. El-Demerdash, A.; Atanasov, A.G.; Horbanczuk, O.K.; Tammam, M.A.; Abdel-Mogib, M.; Hooper, J.N.A.; Sekeroglu, N.; Al-Mourabit, A. and Kijjoa, A., Chemical Diversity and Biological Activities of Marine Sponges of the Genus Suberea: A Systematic Review. *Mar. Drugs*, **2019**. *17*(2), p. 1-14.

186. Hirano, K.; Kubota, T.; Tsuda, M.; Watanabe, K.; Fromont, J. and Kobayashi, J., Ma'edamines A and B, Cytotoxic bromotyrosine alkaloids with a Uinque 2(1H)Pyrazinone ring from sponge *Suberea* sp. *Tetrahedron*, **2000**. *56*, p. 8107-8110.

187. Shaala, L.A.; Bamane, F.H.; Badr, J.M. and Youssef, D.T., Brominated arginine-derived alkaloids from the red sea sponge *Suberea mollis. J. Nat. Prod.*, **2011**. *74*(6), p. 1517-1520.

188. Kurimoto, S.I.; Seino, S.; Fromont, J.; Kobayashi, J. and Kubota, T., Ma'edamines C and D, New Bromotyrosine Alkaloids Possessing a Unique Tetrasubstituted Pyridinium Moiety from an Okinawan Marine Sponge *Suberea* sp. *Org. Lett.*, **2019**. *21*(21), p. 8824-8826.

189. Sharma, G.M. and Burkholder, P.R., Studies on the antimicrobial substances of sponges II. Structure and synthesis of a bromine-containing antibacterial, compound from a marine sponge. *Tetrahedron Lett.*, **1967**. *8*, p. 4147-4150.

190. Debitus, C.; Guella, G.; Mancini, I.; Waikedre, J.; Guemas, J.P.; Nicolas, J.L. and Pietra, F., Quinolones from a bacterium and tyrosine metabolites from its host sponge, *Suberea creba* from the Coral Sea. *J. Mar. Biotechnol.*, **1998**. *6*, p. 136-141.

191. Shaala, L.A. and Almohammadi, A., Biologically active compounds from the Red Sea sponge *Suberea* sp. *Pak. J. Pharm. Sci.*, **2017**. *30*, p. 2389-2392.

192. Kijjoa, A.; Watanadilok, R.; Sonchaeng, P.; Silva, A.M.S.; Eaton, G. and Herz, W., 11, 17-Dideoxyagelorin A and B, New bromotyrosine derivatives and analogs from the marine sponge *Suberea* aff. *praetensa. Z. Naturforsch.*, **2001**. *56c*, p. 1116-1119.

193. Tsuda, M.; Sakuma, Y. and Kobayashi, J., Subereamines A and B, New bromotyrosine alkaloids from a sponge *Suberea* species. *J. Nat. Prod.*, **2001**. *64*, p. 980-982.

194. Carroll, J.; Jonsson, E.N.; Ebel, R.; Hartman, M.S.; Holman, T.R. and Crews, P., Probing sponge-derived terpenoids for human 15-Lipoxygenase inhibitors. *JOC*, **2001**. *66*, p. 6847-6851.

195. Kijjoa, A.; Watanadilok, R.; Sonchaeng, P.; Sawangwong, P.; Pedro, M.; Nascimento, M.S.J.; Silva, A.M.S.; Eaton, G. and Herz, W., Further halotyrosine dderivatives from the marine sponge *Suberea* aff. *praetensa. Z. Naturforsch.*, **2002**. *57c*, p. 732-738.

196. Kijjoa, A.; Watanadilok, R.; Sonchaeng, P.; Puchakarn, S.; Sawangong, P. and Herz, W., Bromotyrosine derivatives from the marine sponge *Suberea* aff. *praetensa. Boll. Mus. Ist. Bio. Univ. Genova*, **2004**. *68*, p. 391-397.

197. Bowden, B.F.; McCool, B.J. and Willis, R.H., Lihouidine, a novel spiro polycyclic aromatic alkaloid from the marine sponge *Suberea* n. sp. (Aplysinellidae, Verongida). *JOC*, **2004**. *69*, p. 7791-7793.

198. Abou-Shoer, M.I.; Shaala, L.A.; Youssef, D.T.A.; Badr, J.M. and Habib, A.-A.M., Bioactive brominated metabolites from the Red Sea sponge *Suberea mollis. J. Nat. Prod.*, **2008**. *71*, p. 1464-1467.

199. Shaala, L.A.; Khalifa, S.I.; Mesbah, M.K.; van Soest, R.W.M. and Youssef, D.T.A., Subereaphenol A, a new cytotoxic and antimicrobial dibrominated phenol from the Red Sea sponge *Suberea mollis. Nat. Prod. Comm.*, **2008**. *3*(2), p. 219-222.

200. Buchanan, M.S.; Carroll, A.R.; Wessling, D.; Jobling, M.; Avery, V.M.; Davis, R.A.; Feng, Y.; Xue, Y.; Oster, L.; Fex, T., et al., Clavatadine A, A natural product with selective recognition and irreversible inhibition of factor XIa. *J. Med. Chem.*, **2008**. *51*, p. 3583-3587.

201. Buchanan, M.S.; Carroll, A.R.; Wessling, D.; Jobling, M.; Avery, V.M.; Davis, R.A.; Feng, Y.; Hooper, J.N.A. and Quinn, R.J., Clavatadines C-E Guanidine alkaloids from the Australian sponge *Suberea clavata. J. Nat. Prod.*, **2009**. *72*, p. 973-975.
202. Shaker, K.H.; Zinecker, H.; Ghani, M.A.; Imhoff, J.F. and Schneider, B., Bioactive metabolites from the sponge *Suberea* sp. *Helv. Chim. Acta*, **2010**. *7*, p. 2880-2887.
203. Xu, M.; Davis, R.A.; Feng, Y.; Sykes, M.L.; Shelper, T.; Avery, V.M.; Camp, D. and Quinn, R.J., Ianthelliformisamines A-C, antibacterial bromotyrosine-derived metabolites from the marine sponge *Suberea ianthelliformis. J. Nat. Prod.*, **2012**. *75*(5), p. 1001-1005.
204. Wright, A.D.; Schupp, P.J.; Schror, J.P.; Engemann, A.; Rohde, S.; Kelman, D.; de Voogd, N.; Carroll, A. and Motti, C.A., Twilight zone sponges from Guam yield theonellin isocyanate and psammaplysins I and J. *J. Nat. Prod.*, **2012**. *75*(3), p. 502-506.
205. Mani, L.; Jullian, V.; Mourkazel, B.; Valentin, A.; Dubois, J.; Cresteil, T.; Folcher, E.; Hooper, J.N.A.; Erpenbeck, D.; Aalbersberg, W., et al., New antiplasmodial bromotyrosine derivatives from *Suberea ianthelliformis Chem. Biodivers.*, **2012**. *9*, p. 1436-1451.
206. Lee, Y.J.; Han, S.; Lee, H.S.; Kang, J.S.; Yun, J.; Sim, C.J.; Shin, H.J. and Lee, J.S., Cytotoxic psammaplysin analogues from a *Suberea* sp. marine sponge and the role of the spirooxepinisoxazoline in their activity. *J. Nat. Prod.*, **2013**. *76*(9), p. 1731-1736.
207. Lee, J.; Shin, A.Y. and Lee, H.-S., Isolation and Synthesis of Luffariellolide Derivatives and Evaluation of Antibacterial Activities against Gram-Negative Bacteria. *Bull. Korean Chem. Soc.*, **2017**. *38*(7), p. 804-807.
208. El-Demerdash, A.; Moriou, C.; Toullec, J.; Besson, M.; Soulet, S.; Schmitt, N.; Petek, S.; Lecchini, D.; Debitus, C. and Al-Mourabit, A., Bioactive Bromotyrosine-Derived Alkaloids from the Polynesian Sponge *Suberea ianthelliformis. Mar. Drugs*, **2018**. *16*(5), p. 1-16.
209. Ciminiello, P.; Fattorusso, E. and Magno, S., Chemistry of Verongida sponges, III. Constituents of a Caribbean *Verongula sp. J. Nat. Prod.*, **1994**. *57*(11), p. 1564-1569.
210. Mierzwa, R.; King, A.; Conover, M.A.; Tozzi, S.; Puar, M.S.; Patel, M. and Coval, S.J., Verongamine, a novel bromotyrosine-derived histamine H3-Antagonist from the marine sponge *Verongula gigantea. J. Nat. Prod.*, **1994**. *57*(1), p. 175-177.
211. Kochanowska, A.J.; Rao, K.V.; Childress, S.; El-Alfy, A.; Matsumoto, R.R.; Kelly, M.; Stewart, G.S.; Sufka, K.J. and Hamann, M.T., Secondary Metabolites from Three Florida Sponges with Antidepressant Activity. *J. Nat. Prod.*, **2008**. *71*, p. 186-189.
212. Galeano, E.; Thomas, O.P.; Robledo, S.; Munoz, D. and Martinez, A., Antiparasitic bromotyrosine derivatives from the marine sponge *Verongula rigida. Mar. Drugs*, **2011**. *9*(10), p. 1902-1913.
213. Hwang, I.H.; Oh, J.; Zhou, W.; Park, S.; Kim, J.H.; Chittiboyina, A.G.; Ferreira, D.; Song, G.Y.; Oh, S.; Na, M., et al., Cytotoxic activity of rearranged drimane meroterpenoids against colon cancer cells via down-regulation of beta-catenin expression. *J. Nat. Prod.*, **2015**. *78*(3), p. 453-461.
214. Kochanowska-Karamyan, A.J.; Araujo, H.C.; Zhang, X.; El-Alfy, A.; Carvalho, P.; Avery, M.A.; Holmbo, S.D.; Magolan, J. and Hamann, M.T., Isolation and Synthesis of Veranamine, an Antidepressant Lead from the Marine Sponge *Verongula rigida. J. Nat. Prod.*, **2020**. *83*(4), p. 1092-1098.
215. Jiso, A.; Kittiwisut, S.; Chantakul, R.; Yuenyongsawad, S.; Putchakarn, S.; Schaberle, T.F.; Temkitthaworn, P.; Ingkaninan, K.; Chaithirayanon, K. and Plubrukarn, A., Quintaquinone, a Merosesquiterpene from the Yellow Sponge *Verongula* cf. *rigida* Esper. *J. Nat. Prod.*, **2020**. *83*(2), p. 532-536.
216. Zwierzyna, M.; Vogt, M.; Maggiora, G.M. and Bajorath, J., Design and characterization of chemical space networks for different compound data sets. *J. Comput. Aided Mol. Des.*, **2015**. *29*(2), p. 113-125.
217. Newman, M.E.J., Networks an Introduction. *Oxford University Press*, **2010**.
218. Blondel, V.D.; Guillaume, J.-L.; Lambiotte, R. and Lefebvre, E., Fast unfolding of communities in large networks. *J. Stat. Mech. Theory Exp.*, **2008**. *2008*(10).
219. Weininger, D., SMILES, a chemical language and information system 1. Introduction to methodology and encoding rules. *J. Chem. Inf. Comput. Sci.*, **1987**. *28*, p. 31-36.
220. Weininger, D., Smiles 3. Depict. Graphical Depiction of Chemical Structures. *J. Chem. Inf. Comput. Sci.*, **1990**. *30*(3), p. 237-243.

221. Zhang, B.; Vogt, M.; Maggiora, G.M. and Bajorath, J., Design of chemical space networks using a Tanimoto similarity variant based upon maximum common substructures. *J. Comput. Aided Mol. Des.*, **2015**. *29*(10), p. 937-950.
